# Tryptophan-Restricted Intermittent Diet Alleviates Estrogen Deficiency-Induced Osteoporosis via Regulating Coupling Effects of “Gut-Bone” Axis

**DOI:** 10.7150/ijbs.128460

**Published:** 2026-04-23

**Authors:** Tingwen Xiang, Shiyu Xiao, Chuan Yang, Dongyang Zhang, Yanghao You, Langlang Xie, Zhiguo Ling, Xiaohua Wang, Chengmin Zhang, Gang Huang, Dong Sun, Yueqi Chen, Fei Luo

**Affiliations:** 1 Department of Orthopedics, Southwest Hospital, Third Military Medical University (Army Medical University), Chongqing 400038, China; 2 Department of Biomedical Materials Science, Third Military Medical University (Army Medical University), Chongqing 400038, China; 3 Institute of Immunology, Third Military Medical University (Army Medical University), Chongqing 400038, China; 4 Department of Biochemistry and Molecular Biology, College of Basic Medical Science, Third Military Medical University (Army Medical University), Chongqing 400038, China; 5 Medical School of Chinese PLA, Beijing 100853, China; 6 Senior Department of Orthopedics, The Fourth Medical Center of Chinese PLA General Hospital, Beijing 100048, China.; 7 National Clinical Research Center for Orthopedics, Sports Medicine and Rehabilitation, Beijing 100048, China.; 8 Department of Orthopedics, Chinese PLA 76th Army Corps Hospital, Xining 810000, China

**Keywords:** osteoporosis (OP), tryptophan-restricted intermittent diet, gut-bone axis, M1 macrophages polarization, osteoclast apoptosis

## Abstract

Osteoporosis (OP) is a chronic and severe skeletal metabolic disorder resulting from excessive bone erosion activity and compromised bone formation. Emerging evidence highlights that the “gut-bone” axis plays a critical role in maintaining bone homeostasis via modulating gut microbiota and relevant metabolites. Individually, diet-derived tryptophan acts as one of the primary environmental factors that modulate the microbiota-bone crosstalk. Nevertheless, the promising modulatory mechanism of the “diet-microbiome-bone” axis remains unknown during OP progression. In this regard, the study focused on the dominant role of tryptophan-restricted intermittent diet during estrogen deficiency-induced OP. Upon these findings, micro-computed tomography (μCT) evaluation and histomorphometric analysis have confirmed that treatment with tryptophan-restricted intermittent diet effectively mitigated bone loss and improved bone microarchitecture. To unravel the underlying mechanism, we performed 16S rDNA gene sequencing and untargeted metabolomics to confirm the alteration of microbial community composition and metabolite profiles. Additionally, biotin was further identified as a significant microbiota-derived metabolite involved in M1 macrophage polarization and mature osteoclast apoptosis when administered with tryptophan-restricted intermittent diet. Thus, we summarized that treatment with tryptophan-restricted intermittent diet could perform a protective function during OP via modulating the coupling effects of gut microbiota and bone homeostasis, which may provide a potential therapeutic strategy for OP.

## Introduction

Osteoporosis (OP) is a pathological condition characterized by reduced bone mineral density (BMD), impaired skeletal microarchitecture, and an elevated risk of fragility fractures. The critical pathological feature reflects a disequilibrium between bone resorption and bone formation. At the cellular level, the imbalance is predominantly driven by heightened differentiation and activation of osteoclasts, where the excessive resorptive function directly contributes to bone loss and structural deterioration 1, 2. The polarization of macrophages toward a pro-inflammatory M1 phenotype within the bone microenvironment constitutes a detrimental factor in bone metabolism. These M1 macrophages secrete several inflammatory cytokines such as TNF-α and IL-6, which actively promote osteoclastogenesis and sustain osteoclast activity, thereby exacerbating pathological bone loss. Beyond established clinical risk factors, such as hormonal imbalance, aging, and genetic predisposition, the gut microbiota, which is the complex community of microorganisms residing in the gastrointestinal tract, has emerged as a pivotal modulator of bone homeostasis 3, 4.

Gut microbiota has been demonstrated to be extensively involved in the pathophysiology of OP through multiple mechanisms, including regulation of the gut barrier, immune modulation, and production of bioactive metabolites 5. On the one hand, the gut microbiota metabolizes dietary fibers and related substrates into short-chain fatty acids (SCFAs) and indole derivatives, which subsequently enter the systemic circulation and directly influence osteoblasts and osteoclasts, ultimately modulating skeletal homeostasis. Concurrently, disruption of the intestinal barrier integrity can lead to the systemic translocation of endotoxins and inflammatory mediators, which further promotes bone loss through enhanced systemic inflammation and direct effects on bone turnover 6, 7. Accumulating evidence indicates that dietary configuration is intimately coupled with gut microbiota composition, thereby exerting a profound impact on host systemic physiology. For example, Mediterranean diets, which are rich in plant-based foods and fiber, have been shown to enhance the abundance of beneficial taxa and improve BMD, whereas Western diets high in saturated fats tend to promote a pro-inflammatory microbial profile and elevate circulating lipopolysaccharide (LPS), thereby exacerbating osteoclast activity and bone loss 4. Within the regulatory network, dietary tryptophan acts as a pivotal microbiota-dependent regulator coordinating the functional homeostasis of the gut-bone axis through immune and metabolic signaling 8. While adequate tryptophan intake has been positively correlated with mental health benefits, including stress mitigation and anxiety alleviation, its physiological influence is complex and context-dependent 9-11. Notably, gut microbiota-derived metabolite indole has recently been observed to promote pro-inflammatory responses implicated in the pathogenesis of rheumatoid arthritis 12. Nevertheless, the role of dietary tryptophan on estrogen deficiency-induced OP is unknown. Therefore, the study was conducted to investigate the causal relationship between tryptophan-restricted intermittent diet and bone metabolism.

Notably, dietary tryptophan has been identified as a critical modulator that profoundly influences the structural composition and ecological dynamics of the gut microbiota 13, including *Akkermansia*, *Lactobacillus*, and *Bifidobacterium*, which generate metabolites that mediate gut-bone crosstalk. Specifically,* Akkermansia* demonstrates positive associations with enhanced intestinal barrier integrity and improved metabolic health 14-16. These bacteria exhibit the potential for the production of biotin (vitamin B7), which is a water-soluble vitamin essential for carboxylase enzymes in cellular metabolism 17-19. However, the specific regulatory mechanisms by which biotin modulates osteoclast activity, macrophage polarization, and the progression of OP remain unclear.

To address these gaps, this study was designed to systematically investigate the effects of a tryptophan-restricted intermittent diet in a murine model of postmenopausal OP. Through integrating 16S rDNA sequencing and untargeted metabolomics on fecal samples from ovariectomy (OVX) mice fed either tryptophan-restricted intermittent diet or normal diet, respectively, we found that intermittent tryptophan restriction could reshape the composition of gut microbiota and alter microbial metabolite profiles, especially enriching *Akkermansia* and *Bifidobacterium* while elevating the concentration of biotin. Further experiments revealed that biotin inhibited M1 macrophage polarization and induced mature osteoclast apoptosis in a mitochondrial-dependent manner, thereby ameliorating bone loss in osteoporotic mice. Summarily, these results proposed a novel dietary strategy for modulating bone homeostasis through gut microbiota remodeling, as well as highlighting biotin as a predominant metabolite for treating OP.

## Materials and Methods

### Mice

All experimental subjects were female C57BL/6J mice at 8 weeks old, sourced from Hunan SJA Laboratory Animal Co., Ltd. Animals were housed under specific pathogen-free (SPF) conditions with a controlled 12-hour light/dark cycle and constant temperature. The present study was approved by the Animal Ethics Committee of Army Medical University (Approval No. AMUWEC20232385), and all procedures were conducted in compliance with national guidelines for ethical animal research. Bilateral OVX was performed aseptically in an SPF environment following anesthesia to establish OP models. After the operation, all experimental mice were randomly divided into the following groups: OVX mice treated with normal diet (OVX-ND), OVX mice treated with tryptophan-restricted intermittent diet (one group named OVX-TRD; another group named TRD), OVX mice under tryptophan-restricted intermittent diet supplemented with 1 g/kg bw/day L-tryptophan (TRD + L-Trp) and additional supplementation of antibiotics (TRD + L-Trp + ABX), OVX mice as a control (OVX-control), and OVX mice treated with biotin (OVX-biotin). Mice under the intermittent tryptophan-restricted diet received the tryptophan-deficient diet for 3 days, followed by standard chow for the next 3 days, which was repeated throughout the 8-week experiment. The OVX-biotin group received continuous gavage of biotin at a dose of 4 mg/kg bw/day for 8 weeks. OVX-control groups received equivalent volumes of PBS via continuous gavage for the same duration as the control.

### Cell culture

Bone marrow-derived macrophages (BMDMs) were isolated from 8-week-old female C57BL/6J mice by flushing the femur and tibia with sterile α-MEM (Gibco) supplemented with 10% FBS (Gibco), 1% penicillin-streptomycin (Meilunbio), and 20 ng/mL recombinant murine M-CSF (ABclonal, Cat#: RP02165). For osteoclast differentiation, BMDMs were cultured for 5 days in α-MEM containing 10% FBS, 50 ng/mL M-CSF, and 100 ng/mL RANKL (ABclonal, Cat#: RP02134), with 10 μM biotin (MedChemExpress, Cat#: 58-85-5) added for 3 days. For M1 polarization, BMDMs were maintained for 5 days in α-MEM with 20 ng/mL M-CSF, then stimulated for 24 h with 100 ng/mL LPS (Solarbio, Cat#: L8880) and 20 ng/mL IFN-γ (ABclonal, Cat#: RP01075), in the presence or absence of 10 μM biotin. IEC-6 intestinal epithelial cells were cultured in DMEM (Gibco). Based on preliminary screening (CCK-8 viability and apoptosis by flow cytometry), 10 μM biotin was selected as the optimal non-toxic dose. All culture media were refreshed every 48 hours throughout the experiments.

### Micro-CT

Femora were excised, fixed in 4 % paraformaldehyde for 48 h, followed by scanning ex vivo using the SkyScan 1272 micro-CT system (Bruker, Kontich, Belgium) at 7 µm isotropic voxel size. After image reconstruction with NRecon v.1.6.10, the trabecular bone starting 1.0 mm proximal to the distal growth plate and extending 4.0 mm proximally was delineated as the region of interest (ROI) in CT-Analyst. Three-dimensional renderings were generated with CTvox v.3.3.1, and BMD, bone volume fraction (BV/TV), bone surface/tissue volume (BS/TV), trabecular thickness (Tb.Th), separation (Tb.Sp), and number (Tb.N), cortical thickness (Ct.Th), cortical bone area (Ct.Ar), and cortical area to trabecular area ratio (Ct.Ar/Tr.Ar) were computed.

### Antibiotic treatment (ABX) experiment

To achieve sustained microbiota depletion, mice in the TRD + L-Trp + ABX groups received a sterile-filtered ABX cocktail by oral gavage for 7 days pre-OVX and every other day thereafter for 8 weeks. The daily dose contained 10 mg/mL Vancomycin (100 mg/kg; Aladdin, Cat#: V421514), 20 mg/mL Neomycin sulfate (200 mg/kg; MedChemExpress, Cat#: HY-B0470), 20 mg/mL Metronidazole (200 mg/kg; Aladdin, Cat#: M109873) and 20 mg/mL Ampicillin Na (200 mg/kg; Aladdin, Cat#: A105483), which were dissolved in sterile water 20.

### ELISA

Serum LPS levels were determined with Mouse LPS ELISA Kits (Ruixin Biotechnology, Cat#: rxj202425M) following the manufacturer's protocol.

### Cell viability assay

BMDMs were isolated from 8-week-old female C57BL/6J mice as previously described. Cells (1 × 10^4^/well) were plated in 96-well plates and treated with 20 ng/mL M-CSF for 5 days. After treatment with biotin for 24 hours, the cells were incubated with 10 µL of CCK-8 reagent (bgbiotech) for 80 minutes. The microplate reader measured absorbance at 450 nm.

### Flow cytometric analysis

The cell apoptosis was detected using the Annexin V-FITC/PI Apoptosis Detection Kit (Meilunbio, Cat#: MA0220). BMDMs were harvested following the designated treatments, washed twice with ice-cold PBS and gently resuspended in 100 μL of 1× Binding Buffer. Subsequently, the cell suspension was stained with 10 μL of Annexin V-FITC and 5 μL of PI for 15 minutes in the dark. After incubation, 400 μL of 1× Binding Buffer was added. The stained cells were analyzed within 1 hour using a flow cytometer (CytoFLEX, USA). Data analysis was performed with FlowJo software. For JC-1, osteoclasts were harvested, washed, and incubated for 20 minutes at 37 °C with JC-1 (Meilunbio, Cat#: MA0338-1) in PBS. After one wash, the cells were kept on ice and immediately analyzed.

### JC-1 staining

Mitochondrial membrane potential (MMP) was evaluated using the fluorescent probe JC-1. Adherent osteoclasts were washed and incubated with JC-1 (Meilunbio, Cat#: MA0338-1) at 37 °C for 20 minutes. Following two washes with PBS, fluorescence signals were captured at 590 nm (red, aggregates) and 530 nm (green, monomers). The ratio of red to green fluorescence intensity was quantified as an indicator of MMP.

### TUNEL staining

Apoptosis in mature osteoclasts derived from mouse BMDMs was detected using a one-step TUNEL assay kit (Meilunbio, Cat#: MA0224) following the manufacturer's instructions. Cells were fixed with 4% paraformaldehyde, permeabilized with 0.3% Triton X-100, and incubated with the TUNEL reaction mixture (containing TdT enzyme and TRITC-dUTP) at 37 °C for 60 min in the dark. After the washing steps, nuclei were counterstained with DAPI, and fluorescence images were acquired using a microscope.

### Transmission electron microscopy (TEM)

Cell samples were primarily fixed in 2.5% glutaraldehyde in PBS (pH 7.4) at 4°C overnight, followed by thorough washing in PBS. Post-fixation was performed with 1% osmium tetroxide for 2 h at 4°C. After PBS rinses, dehydration was carried out through a graded ethanol series (50%, 70%, 80%, 90% and 100%), with an overnight incubation in 70% ethanol. Samples were then transferred to acetone and gradually infiltrated with epoxy resin (Pon812) using acetone-resin mixtures (2:1 and 1:1, v/v), followed by pure resin. After embedding, the resin blocks were polymerized at 65°C for 48 h. Blocks were trimmed to expose the tissue, and ultrathin sections (70 nm) were cut using a diamond knife. Sections were double-stained with uranyl acetate and lead citrate, each for 10 min, and finally examined under a FEI Tecnai G2 Spirit transmission electron microscope.

### Western blot

Following treatment, cells were lysed in ice-cold RIPA buffer containing protease and phosphatase inhibitors. Protein concentrations were determined using the BCA assay, and equal amounts were resolved by 10% SDS-PAGE before transfer to PVDF membranes. After blocking with 5% bovine serum albumin (BSA) at room temperature, membranes were incubated overnight at 4 °C with primary antibodies against primary antibodies, including ZO-1 (1:5000; Proteintech, Cat#: 21773-1-AP), Occludin (1:5000; Proteintech, Cat#: 27260-1-AP), p65 (1:1000; Cell Signaling Technology, Cat#: D14E12), p-p65 (1:5000; ABclonal, Cat#: AP1294), IκBα (1:5000; HUABIO, Cat#: ET1603-6), p-IκBα (1:5000; HUABIO, Cat#: HA722770), CD86 (1:5000; ABclonal, Cat#: A21198), TNF-α (1:500; Bioworlde, Cat#: BS1857), Bax (1:500; Bioworlde, Cat#: BS1030), Bcl-2 (1:5000; HUABIO, Cat#: JF104-8), Apaf-1 (1:500; Bioworlde, Cat#: BS60866), and Cytochrome c (1:500; Bioworlde, Cat#: BS1089). After washing with Tris-buffered saline-Tween (TBST), membranes were incubated for 1 h at room temperature with Multi-rAb™ HRP-Goat Anti-Rabbit Recombinant Secondary Antibody (H+L) (1:5000; Proteintech, Cat#: RGAR001) or Multi-rAb™ HRP-Goat Anti-Mouse Recombinant Secondary Antibody (H+L) (1:5000; Proteintech, Cat#: RGAM001). Subsequent steps were performed according to standard Western blotting protocols.

### qPCR

Total RNA was extracted with TRIzon Reagent (CWBIO, Cat#: CW0580S), reverse-transcribed using an ABScript Neo RT Master Mix for qPCR with gDNA Remover for qPCR (ABclonal, Cat#: RK20433) following the manufacturer's protocol (37 °C 2 min, 55 °C 15 min, 85 °C 5 min). Real-time PCR was performed on a CFX96 Touch system (Bio-Rad) using the 2X Universal SYBR Green Fast qPCR Mix (ABclonal, Cat#: RK21203). Relative expression was calculated by 2^(-ΔΔCt) with Gapdh as the endogenous control (Table S1).

### 16S rDNA sequencing

Fecal pellets were aseptically collected from C57BL/6J mice treated with a normal diet and tryptophan-restricted intermittent diet, respectively, then immediately snap-frozen. Microbial community DNA was extracted from diverse sample matrices via a modified cetyl-trimethylammonium bromide protocol. The integrity and quality of the extracted DNA were assessed by agarose gel electrophoresis, followed by precise quantification. PCR amplification was subsequently performed using the quantified DNA as template. The resulting PCR products were purified using AMPure XT beads (Beckman Coulter Genomics, Danvers, MA, USA) and quantified with the Qubit fluorometric system (Invitrogen, USA). Purified amplicons were further size-selected and refined using the AMPure XT bead-based recovery kit. The quality and concentration of the final sequencing libraries were evaluated using the Agilent 2100 Bioanalyzer (Agilent Technologies, USA) in conjunction with the Illumina library quantification kit (Kapa Biosystems, Wilmington, MA, USA). Paired-end sequencing (2×250 bp) was conducted on the NovaSeq 6000 platform (Illumina, USA) using the NovaSeq 6000 SP Reagent Kit (500 cycles). Raw sequencing reads were demultiplexed. Paired-end sequences were then merged and subjected to quality filtering. Denoising, chimera removal, and length trimming were performed using the DADA2 algorithm via the QIIME2 pipeline (denoise-paired method). The final output included a feature table of amplicon sequence variants (ASVs) and representative sequences. Downstream analyses comprised microbial community assessments (α and β diversity), taxonomic classification against reference databases (SILVA), and statistical identification of differentially abundant ASVs across sample groups.

### Untargeted metabolomics evaluation

Fecal samples collected from mice were subjected to metabolite extraction using a 50% methanol buffer. Chromatographic separation was performed on an UltiMate 3000 UPLC system (Thermo Fisher Scientific, Bremen, Germany) equipped with an ACQUITY UPLC T3 column (100 mm × 2.1 mm, 1.8 μm; Waters, Milford, USA) maintained at 40°C. The mobile phase consisted of solvent A (aqueous solution with 5 mM ammonium acetate and 5 mM acetic acid) and solvent B (acetonitrile). Metabolites eluted from the column were measured through a high-resolution tandem mass spectrometer, Q-Exactive (Thermo Scientific). Raw data were processed with XCMS for peak picking, alignment, and annotation, followed by metabolite identification using CAMERA and metaX. Annotations were validated by matching against the KEGG and HMDB databases. Statistical analyses were conducted via R software, and pathway enrichment analysis was performed using a hypergeometric test.

### RNA transcriptomic sequencing

Total RNA was harvested from cultured cells using TRIzol reagent according to the manufacturer's instructions. RNA quantity and purity were assessed with a Nanodrop-2000 spectrophotometer (Thermo Fisher), and integrity was verified on Agilent 2100, LabChip GX. mRNA was enriched with mRNA Capture Beads, eluted in Tris Buffer at 80 °C, re-bound with Beads Binding Buffer, then fragmented in Frag/Prime Buffer in a thermal cycler. First- and second-strand cDNA were synthesized and purified. The purified product was subsequently treated with an end-repair reagent mixture, and the end-repair reaction was carried out; adapter ligation was performed. The ligation product was then purified to isolate fragments within the desired size range. Finally, the library was amplified via PCR and purified. All purification steps throughout the protocol were executed using VAHTSTM DNA Clean Beads (Cat#: N411-03). The quality control of the final constructed library was assessed using the Qsep-400 bioanalyzer system. The entire workflow was performed according to the specifications of the VAHTS Universal V6 RNA-seq Library Prep Kit for Illumina® (Cat#: NR604-02).

### Histological evaluation

Bilateral hind femora were harvested from mice, fixed in 4% paraformaldehyde, decalcified in 10% ethylenediaminetetraacetic acid (EDTA) for 7 days, and paraffin-embedded. Then these samples were deparaffinized, rehydrated through graded ethanol, and subjected to hematoxylin-eosin (H&E), Masson, or tartrate-resistant acid phosphatase (TRAP) staining.

### Immunohistochemical analysis

Femur sections were blocked with 5% BSA and incubated overnight at 4 °C with primary antibodies against osterix (OSX) (1:200; Servicebio, Cat#: GB151900), osteocalcin (OCN) (1:200; Servicebio, Cat#: GB11233), Bcl-2 (1:100; HUABIO, Cat#: JF104-8), Bax (1:100; Bioworlde, Cat#: BS1030), p-p65 (1:100; ABclonal, Cat#: AP1294), and p-IκBα (1:50; HUABIO, Cat#: HA722770), followed by a 1-hour incubation with corresponding secondary antibodies at room temperature after PBS washes.

### Immunofluorescence analysis

Colonic sections were similarly blocked with 5% BSA and probed overnight at 4 °C with primary antibodies directed at zonula occludens-1 (ZO-1) (1:200; Proteintech, Cat#: 21773-1-AP) and Occludin (1:200; Proteintech, Cat#: 27260-1-AP), followed by 1 h incubation with appropriate secondary antibodies. Femur sections were deparaffinized, antigen-retrieved, blocked, and stained overnight with anti-CD86 (1:100; ABclonal, Cat#: A21198) and anti-F4/80 (1:100; ABclonal, Cat#: A27257). BMDMs on 10 mm coverslips were fixed (4% PFA, 15 min), permeabilized (0.3% Triton X-100, 10 min), blocked (5% BSA, 30 min), and stained overnight at 4 °C with anti-p65 (1:400; Cell signaling, Cat#: D14E12). Following PBS washes, Multi-rAb® CoraLite® Plus 594-Goat Anti-Rabbit Recombinant Secondary Antibody (H+L) (1:200, Proteintech, Cat#: RGAR004) was applied for 1-hour at RT, and nuclei were counterstained with DAPI (Elabscience, Cat#: E-CK-A163).

### Statistical analysis

Data were analyzed in GraphPad Prism 9.5 and are shown as mean ± SD. Two-group comparisons were performed using unpaired two-tailed t-tests with Welch's correction when variances differed (F-test). Microbial taxa were compared using Kruskal-Wallis test, and associations were evaluated by Spearman correlation. *p < 0.05, **p < 0.01, ***p < 0.001, ****p < 0.0001; ns, not significant.

## Results

### Tryptophan-restricted intermittent diet alleviated bone loss in ovariectomy-induced osteoporosis

**Figure 1:**
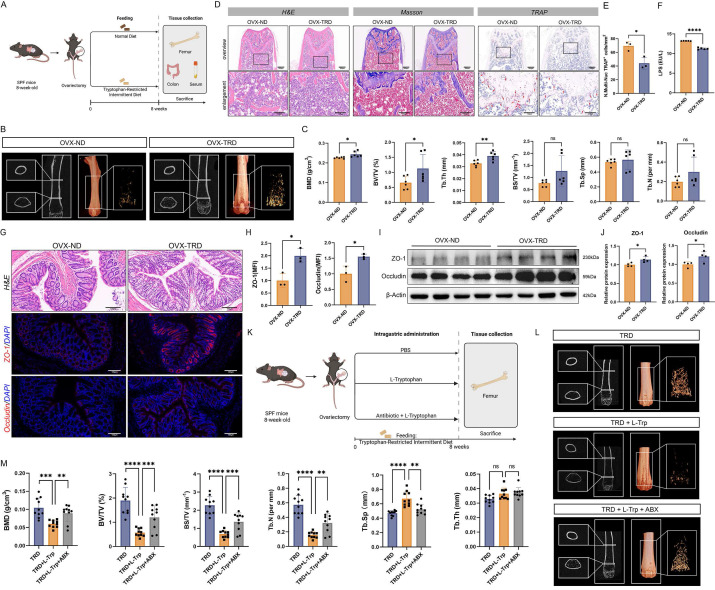
** Intermittent tryptophan restriction mitigated OVX-induced bone loss through microbiota-dependent mechanisms. (A)** Detailed schematic of the animal experimental procedures of the OVX-ND group and OVX-TRD group. **(B)** micro-CT images of femoral longitudinal sections, distal cross-sections, and 3D trabecular structures in the ROI. **(C)** Quantitative analyses of bone histomorphometry indices, including BMD, BV/TV, Tb.Th, BS/TV, Tb.Sp, and Tb.N. **(D)** H&E, Masson, and TARP staining of femur tissue in OVX-ND group and OVX-TRD group. **(E)** Quantitative analysis of the number of TRAP^+^ cells per mm^2^. **(F)** LPS level in mouse serum. **(G)** H&E staining and immunofluorescence staining of ZO-1 and Occludin in colon tissue. **(H)** Mean fluorescence intensity (MFI) quantification of ZO-1 and Occludin after normalization. **(I)** Western blot images of ZO-1 and Occludin in colon tissue from OVX-ND group and OVX-TRD group. **(J)** Relative protein expression levels of ZO-1 and Occludin in colon tissue. **(K)** Detailed schematic of the animal experimental procedures of TRD group, TRD + L-Trp group, and TRD + L-Trp + ABX group. **(L)** micro-CT images of femoral longitudinal sections, distal cross-sections, and 3D trabecular structures in the ROI. **(M)** Quantitative analyses of bone histomorphometry indices, including BMD, BV/TV, BS/TV, Tb.Sp, Tb.N, and Tb.Th.

The OVX-induced OP model has been well established in animal experiments to simulate postmenopausal bone loss 21. In this study, OVX was employed to replicate the pathological features of postmenopausal OP. After OVX, mice were randomly separated into two groups: the OVX group fed with a normal diet (OVX-ND group) and OVX mice receiving the tryptophan-restricted intermittent diet (OVX-TRD group). The tryptophan-restricted intermittent diet was fed to the OVX-TRD group from 0 to 8 weeks, while the control group was provided with normal diet (Figure 1A). Intermittent tryptophan restriction is a dietary intervention strategy characterized by alternating periods of tryptophan deficiency and sufficiency at fixed durations. In the present study, this intervention was implemented as alternating 3-day periods of a tryptophan-deficient diet and 3-day periods of standard chow. As control, the OVX-ND group received the standard chow *ad libitum* continuously. Upon euthanasia, femora were analyzed by µCT. The reconstructed microarchitecture and representative images revealed that the implementation of tryptophan-restricted intermittent diet effectively attenuated OVX-induced bone loss (Figure 1B). Quantitative µCT analysis of femur trabecular bone demonstrated the preservation of bone mass mediated by tryptophan-restricted intermittent diet in OVX mice. The tryptophan-restricted intermittent diet led to significantly elevated BMD (*p <* 0.05), BV/TV (*p <* 0.05), and Tb.Th (*p* < 0.01) (Figure 1C). However, no significant differences were observed in cortical bone parameters, including Ct.Th, Ct.Ar, and Ct.Ar/Tr.Ar (*p* > 0.05) (Figure S1A). Moreover, we performed H&E, Masson, and TRAP staining on histological sections (Figure 1D-E). In addition, as shown in Figure S1B-C, osteoblast function was quantified by immunohistochemistry for OCN and OSX, which independently confirmed that intermittent tryptophan restriction simultaneously augmented osteogenic activity (*p* < 0.05) (Figure S1B-C). Previous studies have reported that LPS could induce bone loss through activating inflammatory signaling, promoting osteoclastogenesis and bone resorption while concurrently suppressing osteoblastic activity 22, 23. Circulating LPS levels in murine serum were quantified, which suggested that the OVX-TRD group exhibited a significant reduction in serum LPS concentration (*p* < 0.0001) (Figure 1F). Given the close association between LPS and intestinal barrier integrity 24, the colon tissues were collected and subjected to H&E and immunohistochemistry staining for ZO-1 and Occludin (*p* < 0.05) (Figure 1G-H). Additionally, we also examined the expression levels of tight junction proteins in colon tissues, which exerted a marked enhancement in intestinal barrier integrity (*p* < 0.05) (Figure 1I-J). All findings indicated that implementing tryptophan-restricted intermittent diet could markedly reverse bone loss and bone microarchitecture impairment in OP.

### Intermittent tryptophan restriction preserved the bone mass through the gut microbiota

Accumulating evidence suggests that dysbiosis of the gut microbiota is a critical factor contributing to alterations in the bone microenvironment and represents a potential pathogenic mechanism of OP. The intestinal microbiome modulates bone remodeling through multiple interconnected pathways, including the regulation of intestinal metabolite absorption, maintenance of immune system homeostasis, and modulation of endocrine function 25-27. To evaluate whether the ameliorative effects of the tryptophan-restricted intermittent diet on OP progression were mediated by gut microbiota, a pseudo-germ-free murine model was established through ABX cocktail treatment in OVX mice. Precisely, OVX mice were randomly separated into three groups: TRD group, TRD + L-Trp group, and TRD + L-Trp + ABX group. L-Trp (1 g/kg bw/day) was supplemented by gavage. ABX cocktails effectively depleted the intestinal microbiota, thereby eliminating the confounding influence on host physiology (Figure 1K). After euthanasia, femora were contoured and dissected for analysis using µCT. As shown in Figure 1L, a significant difference among the three groups has been demonstrated. Quantitative analyses of BMD (*p* < 0.001) and microarchitectural parameters including BV/TV (*p* < 0.0001), BS/TV (*p* < 0.0001), Tb.Sp (*p* < 0.0001) and Tb.N (*p* < 0.0001) confirmed that L-tryptophan supplementation significantly exacerbated OP. Upon the substantial clearance of gut microbiota via ABX treatment, BMD and microarchitectural parameters were significantly enhanced (Figure 1L-M). And cortical bone parameters also showed marked improvement, with significant increases in Ct.Th (*p* < 0.01), Ct.Ar (*p* < 0.05), and Ct.Ar/Tr.Ar (*p* < 0.01). (Figure S1D). Notably, gut microbiota substantial clearance reversed the deleterious effects of L-tryptophan supplementation on bone loss, indicating the microbiota-dependent mechanism. Furthermore, femoral sections were subjected to H&E, Masson and TRAP staining (Figure S1E-H). Histomorphometric analyses of stained sections revealed that L-tryptophan supplementation precipitated trabecular bone destruction and elevated osteoclastic resorption, and concomitant ABX administration could reverse the phenotype. Collectively, these data reveal that the gut microbiota partially mediates the skeletal protective effects of tryptophan-restricted intermittent diet.

### Tryptophan-restricted intermittent diet profoundly improved the diversity profile and reshaped community structure of the gut microbiota

**Figure 2:**
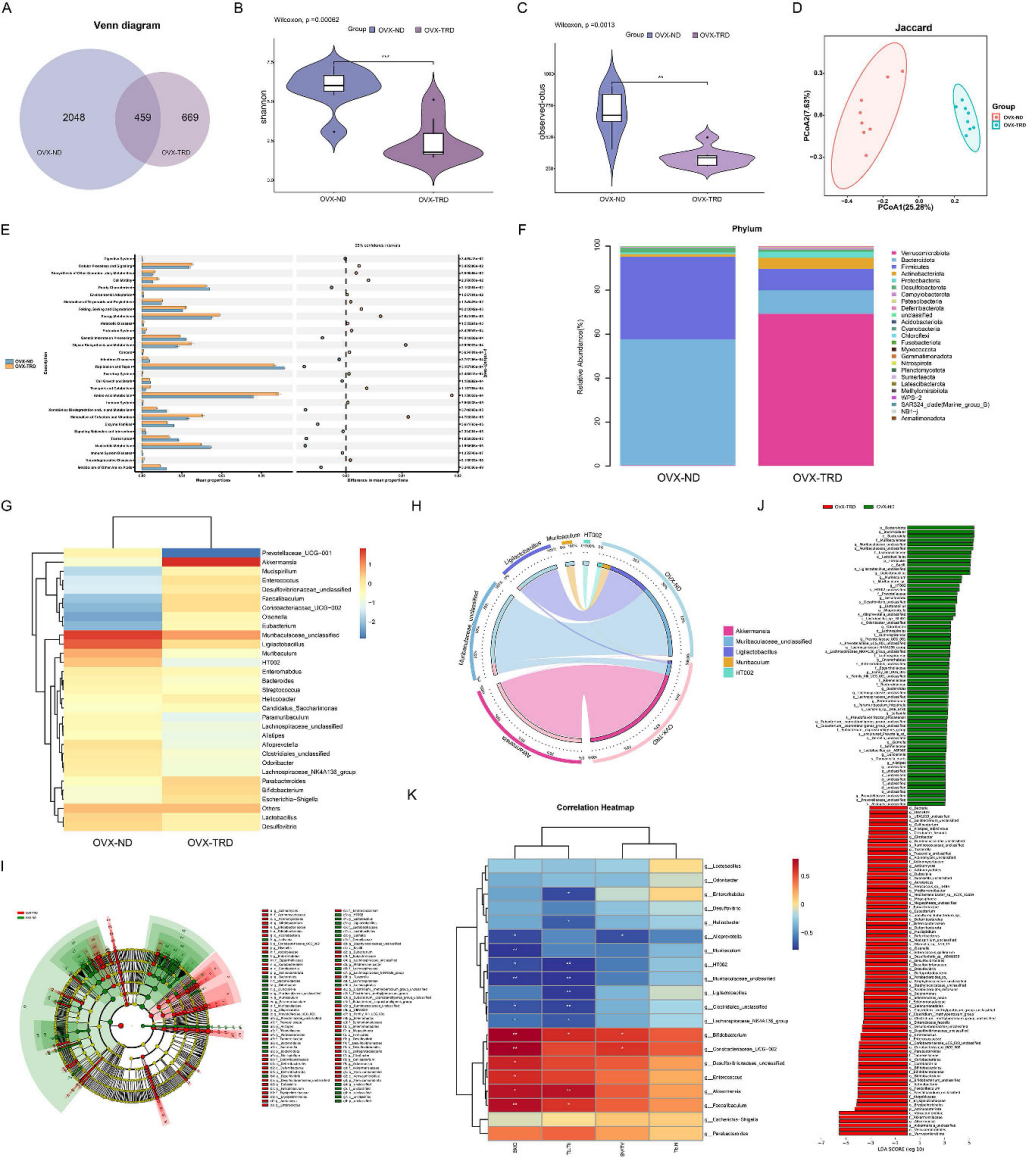
** Intermittent tryptophan restriction altered the composition and architecture of gut microbiota. (A)** Venn diagram of ASVs between OVX-ND group and OVX-TRD group. **(B)** Quantitative analysis of α-diversity based on the Shannon index. **(C)** Quantitative analysis of α-diversity based on the Observed-otus index. **(D)** PCoA based on the Jaccard. **(E)** PICRUSt2-predicted functional profiles using COG database. **(F)** Bar plot of gut microbiota at phylum taxonomic level between OVX-ND group and OVX-TRD group. **(G)** Heatmap of genus-level gut microbiota relative abundance and differential distribution. **(H)** Circos plot of bacterial genus relative abundance. **(I)** Phylogenetic tree generated by LEfSe algorithm demonstrating taxonomic associations between microbiome communities in the OVX-ND group and OVX-TRD group. **(J)** LEfSe cladogram depicting differentially abundant taxa across all ranks between OVX-ND group and OVX-TRD group. **(K)** Heatmap of Spearman's correlation analysis between gut microbial abundance at the genus level and bone parameters.

Given that maintaining tryptophan-restricted intermittent diet ameliorated OP through a gut-microbiota-dependent mechanism, we employed 16S rDNA sequencing to delineate the tryptophan-restricted intermittent diet-induced alterations in microbial community composition and structure. The Venn diagram depicting ASV distribution showed 459 common ASVs between the groups, with the normal diet group containing 2,048 distinctive ASVs and the OVX-TRD group containing 669 unique ASVs (Figure 2A). Thereafter, α-diversity analysis was implemented to quantify species richness and evenness. Rarefaction curves were generated to assess trends in species turnover and to estimate species richness. As shown in Figure S2A, rarefaction curves reached a plateau, evidencing adequate sampling coverage and saturation of taxon detection within the investigated communities (Figure S2A). Chao1, Observed_otus, Shannon, and Simpson indices were further calculated, and their distributions were visualized with violin plots to statistically and visually evaluate the divergence in diversity between the two groups. Collectively, the results revealed that intermittent tryptophan restriction markedly diminished both the diversity and evenness of the gut microbiota relative to the control diet (Figure 2B-C, Figure S2B-C).

To further investigate the species differences in the intestinal microenvironment between the two dietary patterns, β-diversity analysis was employed to assess the variation among fecal samples from the two groups. Principal coordinates analysis (PCoA) performed on unweighted UniFrac, weighted UniFrac, Jaccard, and Bray-Curtis dissimilarity matrices consistently demonstrated a marked divergence in microbial community structure between the two groups (Figure 2D, Figure S2D). Gut microbiota functional profiles were inferred with Phylogenetic Investigation of Communities by Reconstruction of unobserved States 2 (PICRUSt2) against the Clusters of Orthologous Groups (COG) database. A notable increase was observed in the PICRUSt2-predicted abundance of key gene families under tryptophan-restricted intermittent diet such as citrate synthase, transaldolase, and threonine dehydratase (Figure 2E). In addition to predicting the functional capacities of microbial communities, we further employed BugBase to characterize clinically relevant microbiome phenotypes. Marked differences were identified in microbiome-associated phenotypic potentials under the tryptophan-restricted intermittent dietary regimen (Figure S2E).

Based on the relative abundance profiles depicted in Figure 2F, pronounced differences at the phylum level are evident between the two dietary regimens. The conventional diet is dominated by *Bacteroidota*, *Firmicutes*, *Desulfobacterota*, *Actinobacteriota*, and *Proteobacterota*, whereas the OVX-TRD group is primarily populated by *Verrucomicrobiota*, *Bacteroidota*, *Firmicutes*, *Actinobacteriota*, and *Proteobacterota* (Figure 2F). The *Firmicutes*/*Bacteroidota* (F/B) ratio serves as a widely utilized phylum-level indicator of gut microbiota dysbiosis 28. An elevated F/B ratio is frequently correlated with metabolic pathologies such as obesity and type 2 diabetes, reflecting a compositional shift toward increased energy harvest potential 29. In addition to the roles in metabolic disorders, the F/B ratios are also involved in the pathogenesis of inflammatory and autoimmune disorders such as rheumatoid arthritis and inflammatory bowel disease 28, 30. Recent studies in OP models have further demonstrated that modulation of the F/B ratio is associated with significant changes in bone density, thereby implicating the gut microbiota in the regulation of bone homeostasis 31. Assessment of the microbial heatmap indicated that the decrease in *Bacteroidota* was notably more pronounced than that in *Firmicute*s. Additionally, the OVX-TRD group concurrently enriched the intestinal *Verrucomicrobiota*, which has been proposed as a candidate for ameliorating several bone diseases 16, 32. These findings imply that adopting the tryptophan-restricted intermittent diet beneficially restructures the probiotic landscape of the gut microbiota, thereby contributing to the maintenance of skeletal homeostasis.

Thereafter, we systematically quantified the bacterial pedigree across the two experimental groups at the class, order, and family levels. At the class level, the microbial compositional profiles of the two groups diverged (Figure S3A-B). In the control group, *Bacteroidia* and *Bacilli* constituted the dominant classes, whereas in contrast, they dropped to the second and third positions, dominated by *Verrucomicrobiae* in the OVX-TRD group (Figure S3C). At the order level, a significant elevation in *Verrucomicrobiales* and a marked reduction in both *Bacteroidales* and *Lactobacillales* were observed (Figure S4A-B). The tryptophan-restricted intermittent diet contributed to multilevel restructuring of the gut microbiota, characterized at the family level by a significant expansion of *Akkermansiaceae* alongside a sharp contraction of *Muribaculaceae* and *Lactobacillaceae* (Figure S4C-D). Collectively, the intermittent tryptophan restriction orchestrated a systematic shift in the gut microenvironment, resulting in the selective enrichment of taxa that could antagonize OP processes.

Subsequently, we focused on the alterations of genera between the two groups and further discovered specific bacterial strains that tightly correlated with the attenuation of bone loss induced by tryptophan-restricted intermittent diet. At the genus level, relative abundance profiling revealed that the control group was dominated by *Muribaculaceae_unclassified*, *Ligilactobacillus*, *Muribaculum*, *HT002*, and *Lactobacillus*, whereas the tryptophan-restricted intermittent diet drove a community shift toward *Akkermansia*, *Muribaculaceae_unclassified*, *Parabacteroides*, *Bifidobacterium*, and *Faecalibaculum* (Figure 2G). The bubble plot simultaneously depicts genus-level relative abundance and the corresponding phylum-level taxonomy of each taxon. (Figure S5A). Further evolutionary branching tree demonstrated that *Muribaculaceae_unclassified*, *Parabacteroides*, and *Muribaculum* belong to the phylum *Bacteriodota*, while* Ligilactobacillus*, *HT002*, *Faecalibaculum*, and* Lactobacillus* belong to the phylum *Firmicutes*, and* Bifidobacterium* belongs to the phylum *Actinobacteriota*. *Akkermansia*, the dominant genus in the OVX-TRD group, belongs to the phylum *Verrucomicrobiota* (Figure S5B). Sankey plot also visualized distinct directional flows of microbial abundances from the baseline to the endpoint (Figure S5C). Circos plot further confirmed that *Akkermansia* constitutes the dominant genus within the microbial network with tryptophan-restricted intermittent diet (Figure 2H). Specifically, *Akkermansia* is reported to enhance intestinal barrier function and attenuate systemic LPS translocation, contributing to the alleviation of metabolic conditions such as obesity and insulin resistance. It also exerts protective effects against atherosclerosis, inflammatory bowel disease, autism spectrum disorder, and hypertension 33, 34. Besides, *Faecalibaculum* and its derived metabolites exert anti-tumorigenic properties by attenuating neoplastic proliferation and orchestrating intestinal epithelial homeostasis 35, 36. *Bifidobacterium* also promotes epithelial tight-junction integrity, antagonize enteropathogens, and is associated with tumorigenesis inhibition 37. Through expanding the proliferation of these beneficial probiotics, the tryptophan-restricted intermittent diet strengthened microbial balance and competitive exclusion of pathogens, thereby fostering a more stable and symbiotic gut ecosystem conducive to systemic metabolic and immune health.

Based on the confirmed compositional divergence, we next leveraged linear discriminant analysis (LDA), Effect Size (LEfSe), and Cladogram (based on the highest relative abundance difference at each taxonomic level) analyses to dissect the gut microbial signatures underlying the anti-osteoporotic efficacy of tryptophan-restricted intermittent diet (Figure 2I-J). LEfSe analysis revealed that, across multiple taxonomic levels, adopting the tryptophan-restricted intermittent diet significantly enriched *p__Verrucomicrobiota*, *c__Verrucomicrobiae*, *o__Verrucomicrobiales*, *f__Akkermansiaceae*, *g__Akkermansia*, and *s__Akkermansia_unclassified*. In addition, significant differences were observed in the abundance of *p__Actinobacteriota,* representing a key microbial factor through which the tryptophan-restricted intermittent diet contributed to the mitigation of osteoporotic bone loss. Similarly, indicator species analysis was employed to pinpoint differential gut bacterial genera that have the potential to serve as keystone biomarkers (Figure S5D). Summarily, compared with the normal diet group, maintaining tryptophan-restricted intermittent diet reshaped the gut microbiota architecture simultaneously at the phylum, class, order, family, genus, and species levels. The characteristic microbial alteration may mechanistically underlie the anti-osteoporotic protection.

Correlation analysis of gut microbiota and trabecular bone histomorphometric parameters has been further conducted (Figure 2K). *g__Akkermansia* and *g_Bifidobacterium* exhibited a strong positive association with BMD and Tb.Th, while *g__HT002*, *Muribaculaceae_unclassified*, and *Muribaculum* displayed the opposite trend, correlating negatively with BMD and Tb.Th. These results firmly support that the elevated probiotics are intimately implicated in skeletal pathophysiology under the condition of intermittent tryptophan restriction.

### The gut microbiota-derived metabolites composition was altered upon tryptophan-restricted intermittent diet

**Figure 3:**
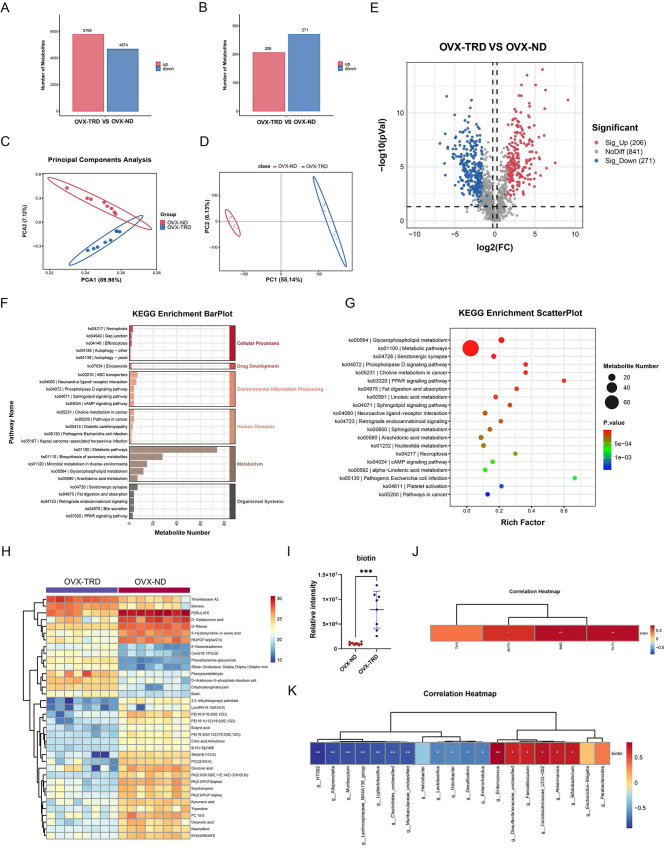
** Tryptophan-restricted intermittent diet reshaped the profile of gut microbiota-derived metabolites. (A)** Statistical plot of differential metabolic ions. **(B)** Statistical plot of differential metabolites. **(C)** PCA of fecal metabolites from OVX-ND group and OVX-TRD group. **(D)** PLS-DA analysis of fecal metabolites from OVX-ND group and OVX-TRD group. **(E)** Volcano plot of metabolomic profiles. **(F)** Bar Chart of KEGG Pathway Enrichment for OVX-ND group and OVX-TRD group. **(G)** Bubble Chart of KEGG Pathway Enrichment. **(H)** Heatmap of differential metabolites in metabolic pathways. **(I)** Comparison of fecal biotin levels between OVX-ND group and OVX-TRD group. **(J)** Heatmap of Spearman's correlation analysis between biotin and bone structural parameters. **(K)** Heatmap of Spearman's correlation analysis between biotin and gut microbiota.

Extensive literature has documented the vital regulatory role of gut microbiota in systemic metabolism. Through 16S rDNA sequencing, it has been revealed that the tryptophan-restricted intermittent diet profoundly reformed the composition and structure of the gut microbiota, which was closely associated with disease progression in osteoporotic mice. To further elucidate the metabolic mediator in the crosstalk between gut microbiota and bone homeostasis, untargeted metabolomics on fecal samples was conducted by liquid chromatography-tandem mass spectrometry (LC-MS/MS), which could comprehensively analyze dynamic changes in intestinal metabolites and uncover complex interactions between microbial activities and host metabolic processes. Based on the quantitative information of processed ions, a total of 22,133 ionic species were identified in the samples, such as Lipids and lipid-like molecules, Organoheterocyclic compounds, Alkaloids and derivatives. Compared with the OVX-ND group, 5,769 metabolites were upregulated in the OVX-TRD group, while 4,674 were downregulated (Figure 3A). Additionally, among the identified secondary metabolites, including Lipids and lipid-like molecules, Organic acids and derivatives, Phenylpropanoids and polyketides, and others, 206 were upregulated and 271 were downregulated in the OVX-TRD group (Figure 3B).

Furthermore, we conducted Principal Component Analysis (PCA) to assess inherent data structure and Partial Least Squares Discriminant Analysis (PLS-DA) to maximize group separation. Both analytical methods consistently demonstrated a clear segregation between the OVX-ND group and OVX-TRD group, indicating that the tryptophan-restricted intermittent diet produced a robust and reproducible metabolic signature (Figure 3C-D). As visualized in the volcano plot, the overall distribution of differential metabolites was further elucidated, exhibiting a clear divide between upregulated and downregulated species (Figure 3E). Consistent with the observation, a total of 206 metabolites were significantly upregulated and 217 were significantly downregulated.

Subsequently, KEGG pathway enrichment analysis was performed on the differentially accumulated metabolites to elucidate the biological implications of the observed metabolic alterations. The analysis identified that the differential metabolites were significantly enriched in KEGG pathways related to Metabolism, Organismal Systems, Environmental Information Processing, Human Diseases, and Cellular Processes (Figure 3F). As shown in Figure 3G, it was observed that the pathways most significantly enriched primarily included Metabolic pathways, Glycerophospholipid metabolism, Serotonergic synapse, phospholipase D signaling pathway, Choline metabolism in cancer, PPAR signaling pathway, and Fat digestion and absorption (Figure 3G). Specifically, the greatest number of metabolites were significantly enriched within the metabolic pathway. Metabolic pathways constitute an evolutionarily conserved, enzyme-driven reaction network that sequentially converts nutritional substrates into ATP, reducing equivalents, biomass precursors, and signaling metabolites, which exhibit extensive crosstalk and regulatory interplay with other cellular processes, collectively ensuring the maintenance of cellular and organismal homeostasis 38, 39. Beyond the canonical catabolism and anabolism of carbohydrates, lipids, proteins, and nucleotides, the pathway simultaneously mediates the *de novo* biosynthesis, salvage, and interconversion of cofactors and vitamins. Thereafter, we analyzed the metabolic pathways to identify differentially expressed metabolites and selected metabolites with the most significant fold-change (FC) values (log_2_|FC|>2.8) (Figure 3H). Further analysis of the metabolic pathway highlighted biotin as a metabolite of significant interest, attributable not merely to its significant differences (*p*<0.001), but also to its intimate association with the gut microbiota and systemic metabolism, which suggests a potential compensatory mechanism (Figure 3I). Biotin, a water-soluble vitamin synthesized de novo by the gut microbiota, is efficiently absorbed across the intestinal epithelium into the systemic circulation. Critically, *Bifidobacterium* has been identified as one of the principal biotin-producing taxa within the human intestine 19, 40, which aligns precisely with previous 16S rDNA gene sequencing data demonstrating significant enrichment of the genus. Accumulating evidence indicates that biotin is indispensable for normal growth and development 41, 42. Given its pivotal role as an essential coenzyme for carboxylases that are involved in crucial processes such as fatty acid synthesis, gluconeogenesis, and amino acid metabolism, biotin may critically enhance the central metabolic pathways, potentially supporting cellular energy production and biosynthetic demands under the experimental conditions. Beyond its classical metabolic functions, biotin also acts as a pleiotropic regulatory molecule, influencing a wide range of cellular activities such as signal transduction, gene expression, cell proliferation, and differentiation 43.

Additionally, Spearman correlation analysis revealed that fecal biotin levels were positively associated with several bone-protective indices, including femoral BMD, BV/TV, and Tb.Th (Figure 3J). At the genus level, the abundance of *Bifidobacterium*, one of the main biotin-producing taxa, exhibited a significant positive correlation with both biotin excretion and BMD, BV/TV and Tb.Th (Figure 2K, Figure 3K). All the above indicate that microbiota-dependent biotin availability was statistically linked to favorable bone microarchitecture, which supported the hypothesis that gut microbial biotin metabolism may modulate systemic bone homeostasis.

### Biotin alleviated osteoporotic bone loss and restored intestinal barrier integrity *in vivo*

**Figure 4:**
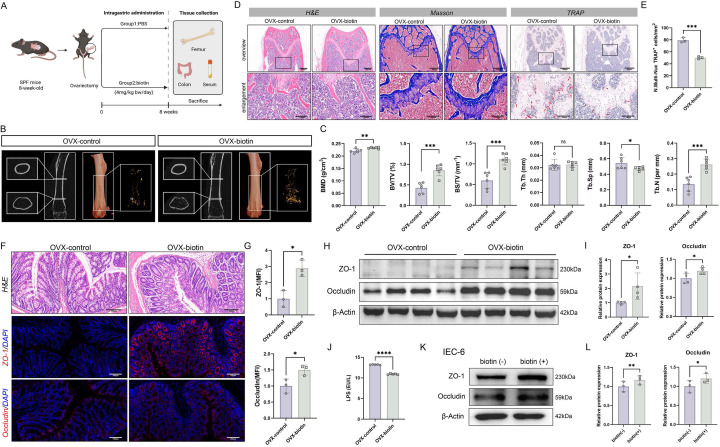
** Biotin prevented bone loss through restoration of intestinal barrier integrity in OVX mice. (A)** Detailed schematic of the animal experimental procedures of OVX-control group and OVX-biotin group. **(B)** micro-CT images of femoral longitudinal sections, distal cross-sections, and 3D trabecular structures in the ROI. **(C)** Quantitative analyses of bone histomorphometry indices, including BMD, BV/TV, BS/TV, Tb.Th, Tb.Sp and Tb.N. **(D)** H&E, Masson, and TARP staining of femur tissue in OVX-control group and OVX-biotin group. **(E)** Quantitative analysis of the number of TRAP^+^ cells per mm^2^. **(F)** H&E staining and immunofluorescence staining of ZO-1 and Occludin in colon tissue. **(G)** MFI quantification of ZO-1 and Occludin after normalization. **(H)** Western blot images of ZO-1 and Occludin in colon tissue from OVX-control group and OVX-biotin group. **(I)** Relative protein expression levels of ZO-1 and Occludin in colon tissue. **(J)** LPS level in mouse serum. **(K)** Western blot images of ZO-1 and Occludin in IEC-6 cells. **(L)** Relative protein expression levels of ZO-1 and Occludin in IEC-6 cells.

To evaluate the osteoprotective properties of biotin *in vivo*, OVX mice were allocated into two groups: one administered biotin (4 mg/kg bw/day) by gavage and the other receiving a matched volume of PBS as the control group. Consistent with the aforementioned protocol, all animals were euthanized for tissue collection after 8 weeks of dietary intervention (Figure 4A). Micro-CT analysis revealed that biotin administration significantly attenuated bone destruction and decelerated OP progression (Figure 4B-C). Histopathological assessment employing both H&E and TRAP staining techniques clearly demonstrated the inhibitory effects of biotin on bone resorption. The histological examination revealed well-preserved trabecular architecture with notably diminished osteoclast activity. TRAP staining further showed substantially decreased osteoclast presence and activity compared to control groups (*p* < 0.001) (Figure 4D-E). However, analysis of cortical bone parameters, including Ct.Th, Ct.Ar, and Ct.Ar/Tr.Ar, showed no significant changes between the experimental groups (Figure S6A). Additionally, immunohistochemistry quantification of OCN and OSX confirmed that biotin supplementation significantly enhanced osteogenic differentiation and bone matrix deposition (Figure S6B-C). From another perspective, biotin treatment restored intestinal barrier function. In intestinal tissue, both immunofluorescence staining and Western blot analyses demonstrated that biotin significantly upregulated the expression of the tight junction proteins Occludin and ZO-1 at the protein level (*p* < 0.05) (Figure 4F-I). As determined by ELISA, it was observed that serum LPS concentration was significantly decreased in the OVX-biotin group relative to controls (*p* < 0.0001) (Figure 4J). In parallel, the protective effect of biotin was corroborated in IEC-6 intestinal epithelial cells. Supplementation of biotin significantly elevated expression of the tight-junction constituents ZO-1 (*p* < 0.01) and Occludin (*p* < 0.05) at the protein levels, indicating a direct reinforcement of intestinal epithelial barrier function, which thereby attenuated paracellular permeability and limited antigenic translocation (Figure 4K-L).

### Gut microbiota-derived biotin restrained macrophage M1 polarization via inhibiting the NF-κB pathway

The alleviative effect of biotin on OP has been clarified through animal experiments, and the study next prioritized elucidating the underlying molecular mechanisms. Bone homeostasis is precisely orchestrated by the dynamic polarization of macrophages, wherein the pro-inflammatory M1 phenotype and the anti-inflammatory M2 phenotype critically regulate the balance between osteoblastic bone formation and osteoclastic bone resorption 44, 45. Disturbed macrophage polarization, especially M1 polarization, drives pathological bone destruction by amplifying osteoclastogenesis via secreting pro-inflammatory cytokines such as TNF-α and IL-1β, and activating the RANKL-RANK pathway, thereby disrupting bone homeostasis toward resorption.

**Figure 5:**
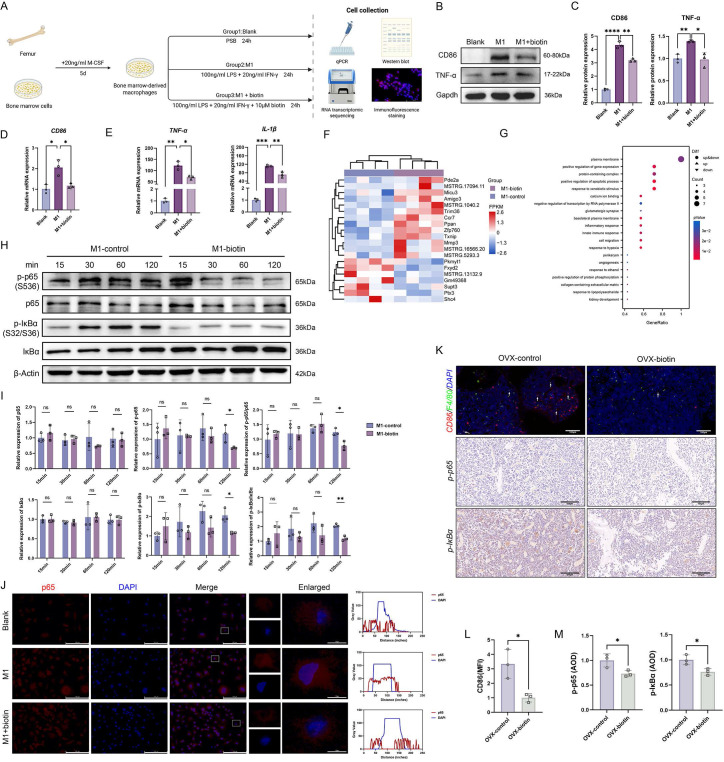
** Gut microbiota-derived biotin restrained macrophage M1 polarization through inhibition of the NF-κB pathway. (A)** Schematic diagram of the *in vitro* experiments. **(B)** Representative Western blot images of CD86, TNF-α and internal control (Gapdh) in BMDMs in the presence or absence of LPS+IFN-γ and biotin. **(C)** Relative protein levels of CD86 and TNF-α were semi-quantitatively analyzed and normalized to Gapdh. **(D)** Relative mRNA expression levels of *CD86*. **(E)** Relative mRNA expression levels of *TNF-α*, *IL-1β*. **(F)** Cluster heatmap of differential expression analysis from RNA sequencing. **(G)** GO enrichment analysis of differentially expressed genes (adjusted p-value<0.05). **(H)** Representative Western blot images of p-p65, p65, p-IκBα and IκBα in the presence or absence of biotin during the stimulation for 15-120 min, with β-Actin as internal control. **(I)** Western blot quantification of p-p65, p65, p-IκBα and IκBα protein levels (normalized to β-actin) and the calculated p-p65/p65 and p-IκBα/IκBα phosphorylation ratios. **(J)** Representative immunofluorescence images of the intracellular location of the NF-κB p65. **(K)** Immunofluorescence staining of femur sections for CD86 (red) and F4/80 (green). Nuclei were counterstained with DAPI (blue). Immunohistochemistry staining of femur sections for p-p65 and p-IκBα in OVX-control group and OVX-biotin group. **(L)** MFI quantification of fluorescence after normalization. **(M)** Average optical density (AOD) quantification of p-p65 and p-IκBα after normalization.

Previous investigations have explicitly demonstrated that M1-polarized macrophages amplify osteoporotic pathogenesis and maintain an inflammatory microenvironment 46. Subsequently, we investigated the impact of biotin on the polarization phenotype of BMDMs following stimulation with standard M1 stimuli (Figure 5A) 47, 48. To determine the optimal non-cytotoxic concentration of biotin for subsequent experiments, BMDMs were treated with a gradient of biotin concentrations (10 nM, 100 nM, 1 μM, 10 μM, and 100 μM). Cell viability was assessed using a CCK-8 assay, and apoptosis was examined by flow cytometry. The results indicated that a concentration of 10 μM biotin showed no significant toxicity and maintained robust cell viability (Figure S6D-F). Then, the Western blot and qPCR demonstrated that biotin significantly suppressed M1 macrophage polarization in both mRNA and protein expressions (Figure 5B-D). Concurrently, qPCR quantification revealed that biotin significantly inhibited the secretion of pro-inflammatory cytokines associated with M1-polarized macrophages in the mRNA expression of *TNF-α* (*p* < 0.05) and *IL-1β* (*p* < 0.01) (Figure 5E). To elucidate the downstream mechanism through which M1 polarization perturbs bone homeostasis, biotin-treated M1 macrophages and parallel M1-polarized controls were subjected to RNA transcriptomic sequencing. Transcriptomic analysis revealed that biotin treatment significantly altered the global gene expression landscape. Gene Ontology (GO) enrichment analysis identified significant overrepresentation of terms related to the positive regulation of protein phosphorylation (*p* < 0.05) and the inflammatory response (*p* < 0.01) (Figure 5F-G). Notably, protein phosphorylation serves as an upstream regulatory event for the NF-κB pathway activation, translating inflammatory cues into signaling cascades that regulate macrophage polarization. Additionally, the NF-κB signaling pathway critically governs the characteristic pro-inflammatory phenotype through multiple coordinated mechanisms, which orchestrate macrophage polarization through a cascade initiated by IκBα phosphorylation and culminating in p65/p50 nuclear translocation 49. To examine whether biotin influences NF-κB signaling, we systematically evaluated key molecular markers including p65, phosphorylated p65 (p-p65), NF-κB inhibitor IκB-α, and phosphorylated IκBα (p-IκBα). Western blot analysis revealed that at the 120-min time point, biotin treatment significantly inhibited the protein expression levels of both p-p65 and p-IκBα compared to the control group (*p* < 0.05). Notably, the phosphorylated-to-total protein ratio of p65 (*p* < 0.05) and IκBα (*p* < 0.01) was also significantly declined, supporting that biotin modulated the activation state of the NF-κB pathway (Figure 5H-I). Following stimulus-induced proteasomal degradation of IκBα, p65 rapidly translocates from the cytoplasm to the nucleus, ultimately licensing the transcriptional response. Immunofluorescence analysis indicated that biotin markedly curtails p65 nuclear translocation, which results in blocking the expression of targeted genes (Figure 5J). Besides, we further verified the inflammatory pathway *in vivo* through staining of femoral tissue sections harvested from OVX mice treated with biotin for 8 weeks. Immunofluorescence staining for CD86 and F4/80 and quantitative analysis of co-expression demonstrated that the mean intensity was significantly lower in the OVX-biotin group compared to controls. Immunohistochemistry analysis consistently further revealed that biotin supplementation downregulated the levels of p-p65 and p-IκBα, thereby corroborating its inhibitory effect on M1 macrophage polarization (*p*<0.05) (Figure 5K-M). These results delineated a pivotal regulatory mechanism whereby biotin targeted the upstream IκBα degradation and downstream p65 activation in the NF-κB signaling.

### Biotin promoted apoptosis of mature osteoclasts in a mitochondrial-dependent manner

**Figure 6:**
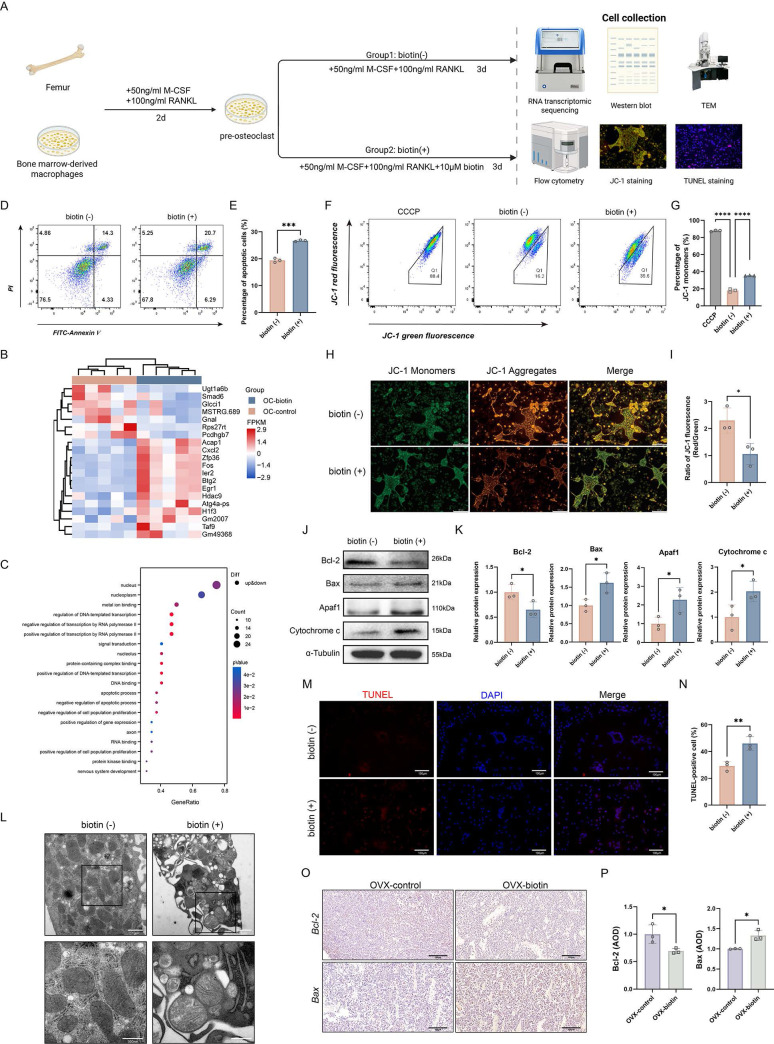
** Biotin triggered apoptosis of mature osteoclasts via the mitochondrial pathway. (A)** Schematic diagram of the *in vitro* experiments. **(B)** Cluster heatmap of differential expression analysis from RNA sequencing. **(C)** GO enrichment analysis of differentially expressed genes (adjusted p-value<0.05). **(D)** Flow cytometry analysis of apoptosis. **(E)** Quantification of apoptotic cells. **(F)** Flow cytometric analysis of JC-1 staining showed a significant shift in the ratio of aggregate to monomer fluorescence in biotin-treated osteoclasts. **(G)** Quantification of the percentage of JC-1 monomers. **(H)** Representative JC-1 fluorescence images showed a shift from red to green fluorescence after treatment with biotin. **(I)** Quantitative analysis of the fluorescence intensity ratio. **(J)** Representative Western blot images showing protein levels of Bcl-2, Bax, Apaf1, and Cytochrome c. α-Tubulin was used as an internal control. **(K)** Relative protein levels were semi-quantitatively analyzed and normalized to α-Tubulin. **(L)** Morphological assessment of osteoclast mitochondria by TEM. **(M)** Representative fluorescence images of osteoclasts stained with DAPI (blue, nuclei) and TUNEL (red). **(N)** Percentage of TUNEL-positive cells. **(O)** Immunohistochemistry staining of femur sections for Bcl-2 and Bax in OVX-control group and OVX-biotin group. **(P)** AOD quantification of Bcl-2 and Bax after normalization.

Our previous studies have already illustrated the beneficial impact of biotin on skeletal homeostasis through an anti-inflammatory manner, demonstrating that biotin alleviated bone resorption by suppressing M1 macrophage polarization and attenuating inflammatory responses. To further investigate the potential direct effects of biotin on osteoclasts, which directly execute bone resorption, the study turned its focus to the survival of osteoclasts treated with biotin (Figure 6A). Firstly, RNA transcriptomic sequencing analysis was performed to compare the transcriptomes of osteoclasts treated with biotin with the untreated group. Our transcriptomic sequencing identified significant alterations in gene expression following biotin treatment, with subsequent GO enrichment analysis revealing a significant association between biotin treatment and the apoptotic process (*p*<0.05). Within the negative regulation of apoptotic process and apoptotic process, a mixed pattern of gene expression was observed including both upregulation and downregulation (Figure 6B-C). According to the results, our subsequent research further focuses specifically on apoptosis in mature osteoclasts. Flow cytometry was used to measure the consequence of biotin on the mature osteoclast apoptosis rate, which demonstrated that apoptotic cells were significantly increased with 10 μM biotin (*p* < 0.001) (Figure 6D-E). Apoptosis is an ATP-dependent, genetically programmed form of regulated cell death indispensable for the maintenance of cellular homeostasis and tissue integrity. Mature osteoclast apoptosis selectively removes senescent or hyper-activated cells, attenuating bone resorption, sustaining skeletal homeostasis, and preventing the exacerbation of osteolytic diseases. Intrinsic apoptosis, also termed the mitochondrial pathway, is a form of programmed cell death centrally regulated by the assembly of the mitochondrial apoptosome 50. Bax is a pro-apoptotic protein that counteracts the cytoprotective action of Bcl-2.

An elevated Bax/Bcl-2 ratio promotes mitochondrial outer-membrane permeabilization, leading to the release of cytochrome c into the cytoplasm. Within the cytosol, cytochrome c binds to apoptotic protease-activating factor 1 (Apaf-1), initiating the formation of the mitochondrial apoptosome. Specifically, in the presence of dATP/ATP, cytochrome c and Apaf-1 oligomerize to form a heptameric apoptosome complex, which recruits and activates initiator caspase-9, triggering the executive caspase cascade and ultimately leading to programmed cell death 51. Extensive studies have demonstrated that mitochondrial membrane permeability increases during the early stages of apoptosis. The collapse of MMP is recognized as one of the earliest events in the apoptotic cascade 52. JC-1, a widely used fluorescent probe, enables sensitive detection of MMP dynamics in apoptotic cells. Using flow cytometry, alterations in mitochondrial fluorescence intensity following biotin treatment were observed, which indicated that the percentage of JC-1 monomers was significantly elevated (*p* < 0.0001) (Figure 6F-G). As shown in Figure 6H, JC-1 staining revealed that biotin-treated osteoclasts exhibited a substantial decrease in red fluorescence intensity compared to the control group. Quantitative analysis confirmed a significant reduction in the red/green fluorescence ratio (JC-1 aggregates/monomers) in the biotin-treated group, indicating a loss of MMP and suggesting the onset of early apoptosis (*p* < 0.05). Carbonyl cyanide m-chlorophenyl hydrazone (CCCP) was used as a positive control to induce mitochondrial depolarization (Figure 6H-I). Moreover, we further examined whether biotin curtailed bone resorption by triggering the apoptotic signaling cascade. Western blot analysis was conducted to examine the expression of Bcl-2, Bax, Apaf-1, and Cytochrome c. As shown in Figure 6J-K, compared with controls, biotin markedly downregulated Bcl-2 while concomitantly elevating Bax expression. Consistent with this, Apaf-1 and Cytochrome c were significantly increased (*p* < 0.05) (Figure 6J-K). Ultrastructural examination via TEM revealed pronounced mitochondrial abnormalities, including swelling, loss of cristae integrity, and the presence of fragmented mitochondria, which are characteristic morphological features of the intrinsic apoptotic pathway (Figure 6L). Further evidence of apoptosis was provided by TUNEL staining, which showed a markedly higher percentage of TUNEL-positive cells in the biotin-treated group (*p* < 0.05) (Figure 6M-N). Consistent with *in vitro* findings, these alterations in protein expression were similarly confirmed in animal experiments. Immunohistochemical analysis of Bcl-2 and Bax also demonstrated a significant increase in expression within femoral bone tissue (*p* < 0.05), indicating that biotin treatment significantly promoted mature osteoclast apoptosis in the bone marrow cavity, leading to suppressed bone resorption (Figure 6O-P). The findings above confirmed biotin-mediated modulation of osteoclast activity via mitochondrial-associated apoptosis, specifically, restraining Bcl-2 expression and enhancing the interaction of Apaf-1 and Cytochrome c.

## Discussion

**Figure 7:**
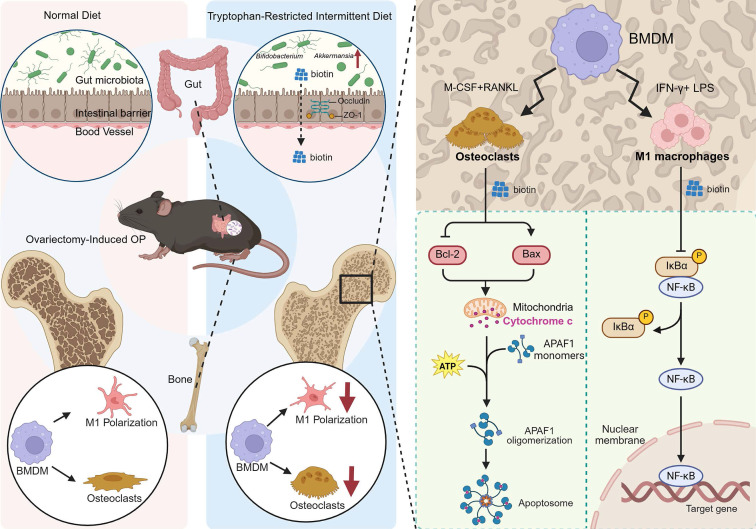
** Schematic for tryptophan-restricted intermittent diet-mediated protective effects on OP progression.** Tryptophan-restricted intermittent diet reshaped the gut microbiota structure and metabolite composition, elevated the level of biotin, which inhibited macrophage M1 polarization and promoted mature osteoclast apoptosis, ultimately ameliorating OP.

The gut-bone axis represents a dynamic and bidirectional communication network. Extensive literature confirms that specific probiotic strains and microbial metabolites directly influence bone remodeling by modulating immune responses, enhancing intestinal barrier integrity, and regulating the activity of osteoclasts and osteoblasts 53-55. Dietary components serve as fundamental substrates for bone matrix synthesis, which could also exert a profound influence on bone remodeling by targeting gut microbial composition and activity. Specific dietary components are metabolized by the gut microbiota into various bioactive compounds, while some nutrients can remodel the microbial community, strengthen the gut barrier, and suppress systemic inflammation, indirectly influencing skeletal homeostasis. Despite the established role of the gut-bone axis in skeletal homeostasis, the microbial signals involved in the crosstalk and the downstream pathways have still not been identified. Herein, we demonstrated that implementing the tryptophan-restricted intermittent diet conferred significant protection against OVX-induced bone loss in mice, which was partially orchestrated by remodeling the gut microbiota and elevating the microbial metabolite biotin. Mechanistically, biotin exerted the osteoprotective effect mediated through suppressing M1 macrophage polarization via inhibition of the NF-κB pathway and directly inducing mature osteoclast apoptosis via the mitochondrial Bcl-2/Bax pathway, ultimately leading to preserved bone mass and microarchitecture (Figure 7). The research significantly advanced our understanding of the gut-bone axis and highlighted the potential of nutritional interventions, such as tryptophan modulation or biotin supplementation, and microbial-based therapies as innovative strategies for the prevention and treatment of postmenopausal OP.

Dietary intermittent tryptophan restriction has a profound impact on the gut microbial ecosystem. 16S rDNA sequencing revealed that tryptophan-restricted intermittent diet substantially remodeled the gut microbiota composition in osteoporotic mice. The observed decline in α-diversity following tryptophan restriction reflected not merely a random loss of species but a deterministic pruning process that reconfigured the gut ecosystem into a highly efficient consortium, which was predominantly composed of *Verrucomicrobiota*, *Actinobacteriota*, and *Firmicutes*. As predicted by PICRUSt2, the compositional restructuring in the gut microbiota was accompanied by a significant functional reprogramming. The upregulation of citrate synthase and threonine dehydratase modules has the potential to enlarge the TCA overflow metabolite pool and increase the availability of reducing equivalents, thereby conferring nutritional and energetic advantages on butyrate-producing taxa and mucin-degrading specialists. Collectively, these changes contributed to the fortification of the epithelial barrier and a reduction in systemic LPS translocation. Notably, tryptophan-restricted intermittent diet exerted a suppressive effect on *HT002* and *Ligilactobacillus*, which possessed genomes that encode indole-3-aldehyde, an aryl-hydrocarbon-receptor agonist that was associated with Th17 differentiation and osteoclastogenesis 56. Through LEfSe and indicator species analyses, *p__Verrucomicrobiota* and *s__Akkermansia_unclassified* have been robustly identified as a well-defined set of biomarkers. The relative abundances of these biomarkers can partially explain the enhancement in trabecular BV/TV. Untargeted LC-MS/MS profiling of fecal extracts detected 22,133 ion features, of which 5,769 were upregulated and 4,674 were downregulated under tryptophan-restricted intermittent diet relative to a normal diet. Among 1,460 annotated secondary metabolites, 206 increased and 271 decreased. PCA and PLS-DA demonstrated a clear segregation between dietary groups, and volcano plots further visualized the significantly regulated metabolites. KEGG pathway enrichment analysis showed that the differentially accumulated metabolites were significantly enriched in multiple metabolic categories, with the general “metabolic pathways” map containing the greatest number. Biotin was identified as a pivotal metabolite whose elevated fecal abundance may partially mediate the osteoprotective effects of tryptophan-restricted intermittent diet.

In fact, dietary factors are regarded as a fundamental environmental determinant that profoundly influences systemic physiology and tissue homeostasis, representing a continuously evolving and biologically meaningful area of investigation 57. Amino acid-based dietary interventions exert dual and complex effects on human metabolism. The pathogenic or therapeutic implications of dietary restriction are determined by a multi-level interplay involving the specific disease pathology, the host's metabolic state, and immune and metabolic cascades driven by the microbiota. Emerging evidence indicates that restricting specific essential amino acids can confer therapeutic benefits. For instance, dietary limitation of certain amino acids has been shown to enhance tumor therapy efficacy 58, 59, while branched-chain amino acid restriction offers a novel paradigm for Alzheimer's disease treatment 60. Conversely, previous literature documents the positive metabolic effects of tryptophan. In this context, the intermittent tryptophan restriction protocol employed in the present study demonstrated osteoprotective effects through profoundly reshaping the gut microbial ecosystem, altering community composition, metabolic output, and functional capacity, suggesting that the influence of gut microbiota and their metabolites on systemic homeostasis may, to some extent, outweigh the effects of tryptophan itself. This dietary-microbial interface constitutes a critical regulatory nexus through which nutritional interventions exert broad physiological effects, with implications that extend far beyond the direct availability of individual amino acids.

Gut barrier integrity prevents the systemic translocation of bacterial products, thereby limiting LPS entry and containing low-grade inflammation that would otherwise promote osteoclast differentiation. Additionally, beyond barrier function and metabolite-mediated signaling, extracellular vesicles have emerged as critical mediators of inter-organ communication, which are derived from intestinal epithelial cells or gut microbiota and serve as novel mediators, delivering microRNAs and proteins to bone marrow targets and enabling precise remote regulation of skeletal remodeling 61-63. For instance, extracellular vesicles secreted by *Akkermansia muciniphila* could translocate to bone tissue to enhance osteogenic activity and inhibit osteoclast formation 62. Glucocorticoid-induced osteonecrosis of the femoral head is associated with depletion of gut* Lactobacillus animalis* and its extracellular vesicles. Under physiological conditions, these vesicles translocate to the femoral head and promote angiogenesis, osteogenesis, and cell survival 61. Importantly, these mechanistic paradigms extend beyond skeletal regulation. Analogous regulatory principles operate along the gut-liver, gut-brain, gut-kidney, and gut-lung axes, wherein diet-driven microbial shifts, barrier function, and vesicular trafficking collectively influence distant organ physiology 64-66. Recent evidence demonstrates that commensal *Bifidobacterium*-derived extracellular vesicles modulated anti-PD-1 immunotherapy efficacy in non-small cell lung cancer by delivering bioactive cargo to tumor cells and enhancing CD8+ T cell infiltration 65. Similarly, gut microbiota-derived outer membrane vesicles translocated across the leaky intestinal barrier into the kidney and directly induce tubulointerstitial inflammation via LPS-caspase-11 pathway activation in diabetic kidney disease 67. Moreover, as previously mentioned, dietary regimens also exert systemic effects by remodeling the gut ecosystem and its inter-organ communication networks 4. Within this paradigm, the gut-vascular axis has been identified as a critical pathway linking nutritional interventions to cardiovascular outcomes. Alternate-day fasting attenuates vascular calcification by enriching *Akkermansia muciniphila* and its extracellular vesicles, which deliver the protein B2URF3 to vascular smooth muscle cells, thereby suppressing osteogenic differentiation and calcification 15. Thus, gut-extraintestinal communication exhibits extensive crosstalk and synergistic interactions among multiple regulatory pathways, collectively constituting a highly integrated and networked regulatory system. Understanding how this networked regulatory system integrates diverse dietary and microbial signals to coordinate multi-organ responses remains an important frontier for future investigation.

Meanwhile, the study has several limitations. First, while the correlative data are strong, definitive causation remains to be established. Causal proof that the specific increase in *Akkermansia* and *Bifidobacterium* is necessary for the observed effects would require fecal microbiota transplantation (FMT) experiments or utilizing germ-free mice recolonized with these specific strains. Notably, it becomes indispensable to identify the specific bacterial strains that fundamentally mediate osteoprotective effects. Secondly, the translational relevance of our findings is constrained by the use of a murine model. Future clinical studies are essential to determine the correlations among gut microbiota composition, biotin levels, and bone mass in postmenopausal women with OP. Furthermore, the safety and feasibility of both tryptophan-restricted intermittent diets and biotin supplementation require further validation. While it is an essential cofactor for mitochondrial carboxylases, supraphysiological doses have been shown to induce mitochondrial dysfunction in osteoclasts. Therefore, identifying the threshold concentration that separates physiological requirement from pharmacological toxicity is a key experimental priority. Besides, given the pivotal role of gut microbiota in mediating dietary effects on bone homeostasis, we should prioritize determining the optimal daily intake of tryptophan, which has strong potential to influence OP development. In addition, specific alterations in gut microbial composition may represent underlying pathophysiological changes in bone metabolism. Probiotics showed promise in improving BMD and trabecular microstructure, demonstrating strong therapeutic potential 68. Whether the combined administration of probiotics and prebiotics yields superior efficacy compared to TRD monotherapy remains a critical and promising question that requires rigorous validation through large-scale clinical trials. Consequently, profiling gut microbiota and measuring biotin concentrations in blood and stool could represent innovative strategies for evaluating OP risk and therapeutic efficacy.

In conclusion, the results reinforced the promise of dietary intervention for metabolic disorders via gut microbiota modulation. We have delineated a novel gut-bone axis in which reduced dietary tryptophan expanded commensal probiotics that synthesize biotin, which repressed NF-κB-dependent M1 macrophage polarization while simultaneously trigged Bcl-2/Bax-mediated mitochondrial apoptosis in mature osteoclasts, thereby alleviating bone loss. Future directions could be dedicated to precision medicine approaches to address patient-specific microbiological and nutritional factors, thereby advancing the overall management of OP.

## Supplementary Material

Supplementary figures and tables.

## Data Availability

The data that support the findings of this study are available within the article and its supplementary materials. Raw data are available from the corresponding authors upon reasonable request.

## Abbreviations

AOD: average optical density
ASV: amplicon sequence variant
ABX: antibiotic
Apaf-1: apoptotic protease-activating factor 1
BSA: bovine serum albumin
BMD: bone mineral density
BS/TV: bone surface/tissue volume
BV/TV: bone volume fraction
BMDMs: bone marrow-derived macrophages
CCCP: carbonyl cyanide m-chlorophenyl hydrazone
COG: Clusters of Orthologous Groups
Ct.Th: cortical thickness
Ct.Ar: cortical bone area
Ct.Ar/Tr.Ar: cortical area to trabecular area ratio
LEfSe: Effect Size
FMT: fecal microbiota transplantation
FC: fold-change
LDA: linear discriminant analysis
LPS: lipopolysaccharide
LC-MS/MS: liquid chromatography-tandem mass spectrometry
MFI: mean fluorescence intensity
MMP: mitochondrial membrane potential
ND: normal diet
OCN: osteocalcin
OP: osteoporosis
OSX: osterix
OVX: ovariectomy
PLS-DA: Partial Least Squares Discriminant Analysis
PICRUSt2: Phylogenetic Investigation of Communities by Reconstruction of unobserved States 2
PCA: Principal Component Analysis
PCoA: Principal coordinates analysis
SCFAs: short-chain fatty acids
SPF: specific pathogen-free
Tb.N: trabecular number
Tb.Sp: trabecular separation
Tb.Th: trabecular thickness
TBST: Tris-buffered saline-Tween
TEM: Transmission electron microscopy
TRD: tryptophan-restricted intermittent diet
Trp: tryptophan
ZO-1: zonula occludens-1
