# Supplementary material for: Tryptophan-Restricted Intermittent Diet Alleviates Estrogen Deficiency-Induced Osteoporosis via Regulating Coupling Effects of “Gut-Bone” Axis
Source: Int J Biol Sci. 2026 Apr 23;22(9):4784–805. doi: 10.7150/ijbs.128460 (PMC13182557; doi:10.7150/ijbs.128460)
Supplement: Supplementary file 1 — Supplementary figures and tables. [file ijbsv22p4784s1.pdf]

Supplementary materials

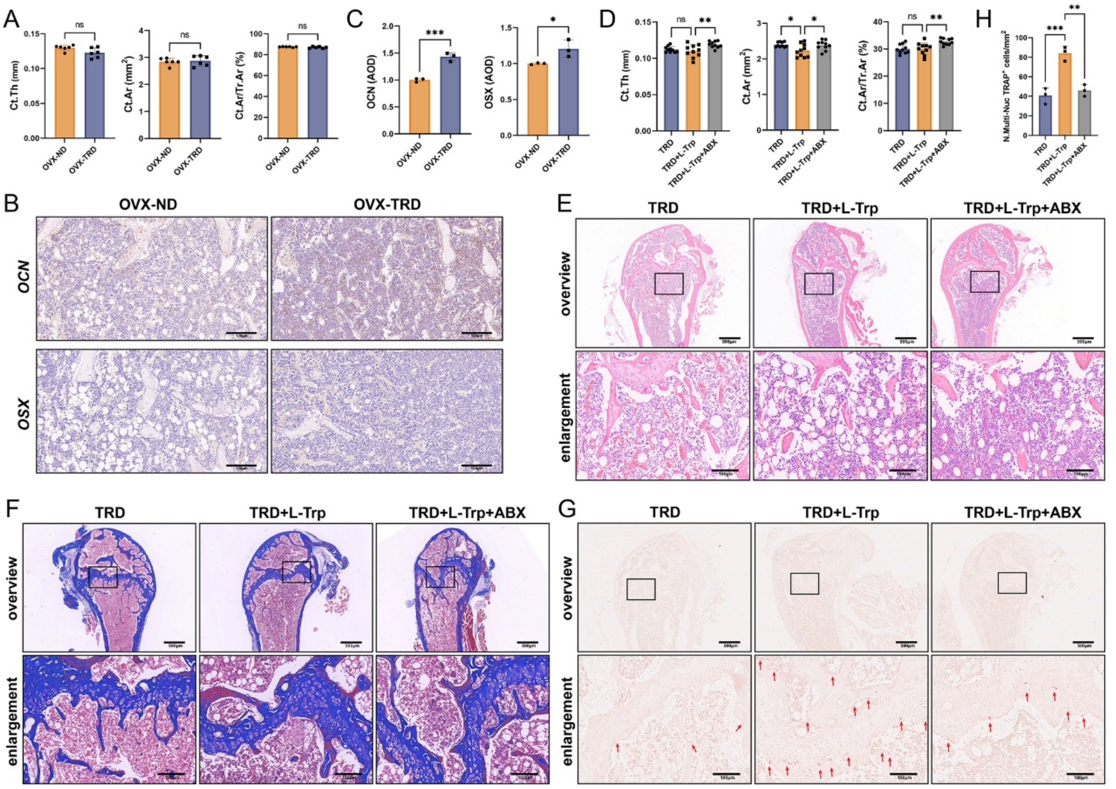

Figure S1: Intermittent tryptophan restriction exerted an osteoprotective effect involved by the gut microbiota. (A) Quantitative analyses of cortical bone histomorphometry indices including Ct.Th, Ct.Ar, and Ct.Ar/Tr.Ar in OVX-ND group and OVX-TRD group. **(B)** Immunohistochemistry staining of femur sections for OSX and OCN. **(C)** AOD quantification of OSX and OCN after normalization. **(D)** Quantitative analyses of cortical bone histomorphometry indices including Ct.Th, Ct.Ar, and Ct.Ar/Tr.Ar among TRD group, TRD + L-Trp group, and TRD + L-Trp + ABX group. **(E-G)** H&E, Masson, and TARP staining of femur tissue in TRD group, TRD + L-Trp group, and TRD + L-Trp + ABX group. **(H)** Quantitative analysis of the number of $TRAP^+$ cells per mm^2.

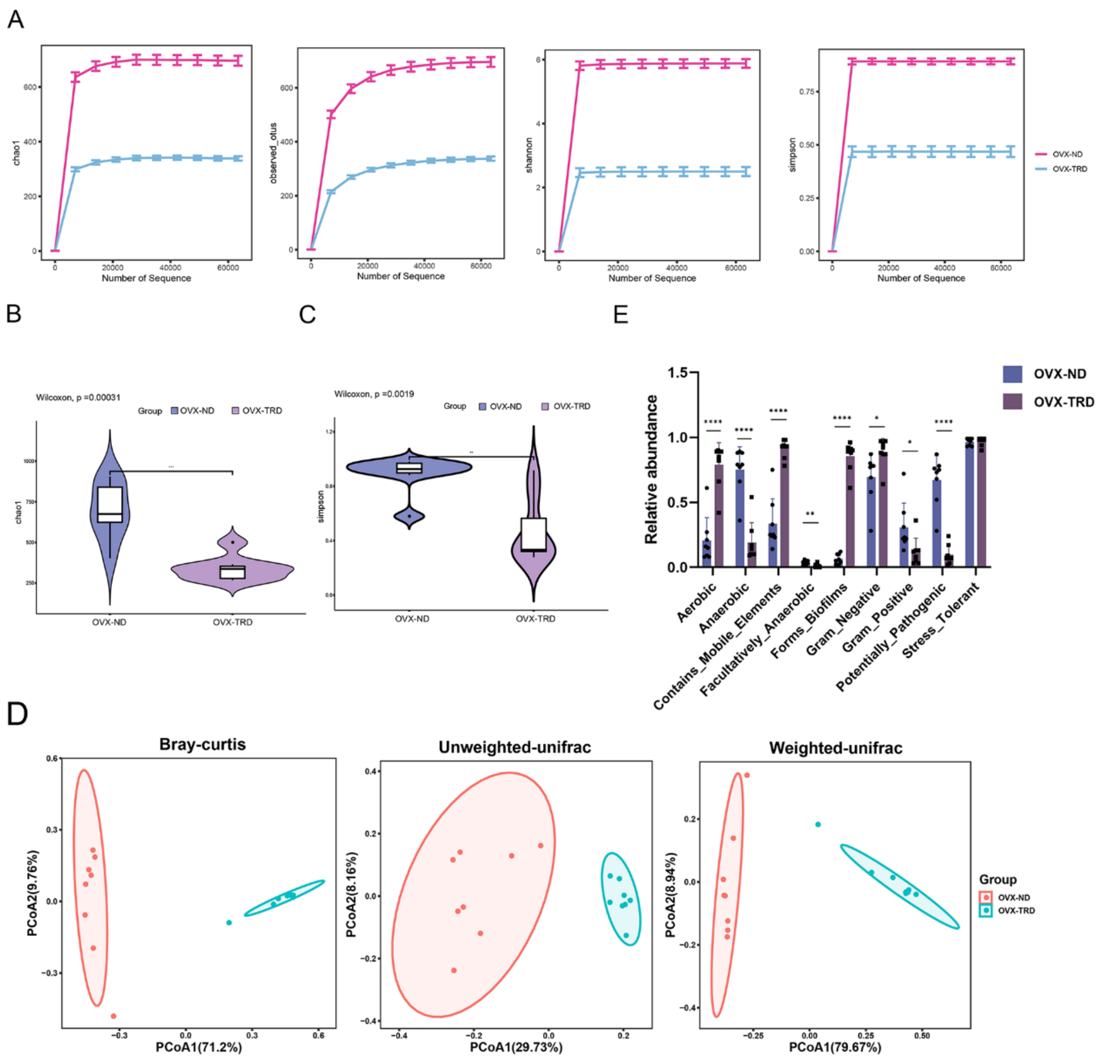

Figure S2: Comprehensive characterization of gut microbiota diversity and composition in the OVX-ND and OVX-TRD groups. (A) Rarefaction curves derived from α-diversity. **(B-C)** Violin plot of related α-diversity consisting of Chao1 and Simpson. **(D)** PCoA based on the Bray-Curtis, Unweighted UniFrac and Weighted UniFrac analyses. **(E)** Bar plot about the abundance of microbiota.

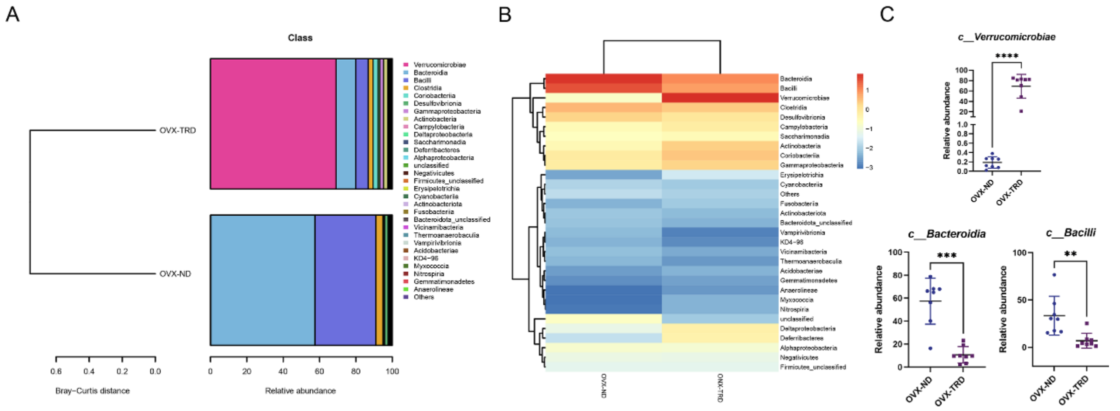

Figure S3: Intermittent tryptophan restriction shifted gut microbiota composition at the class taxonomic level. (A) Bar graph of the bacteria at the class taxonomic level. **(B)** Heatmap depicting different gut microbiota at the class taxonomic level. **(C)** The relative abundance of *c__Verrucomicrobiaea*, *c__Bacteroidia*, and *c__Bacilli* between OVX-ND group and OVX-TRD group.

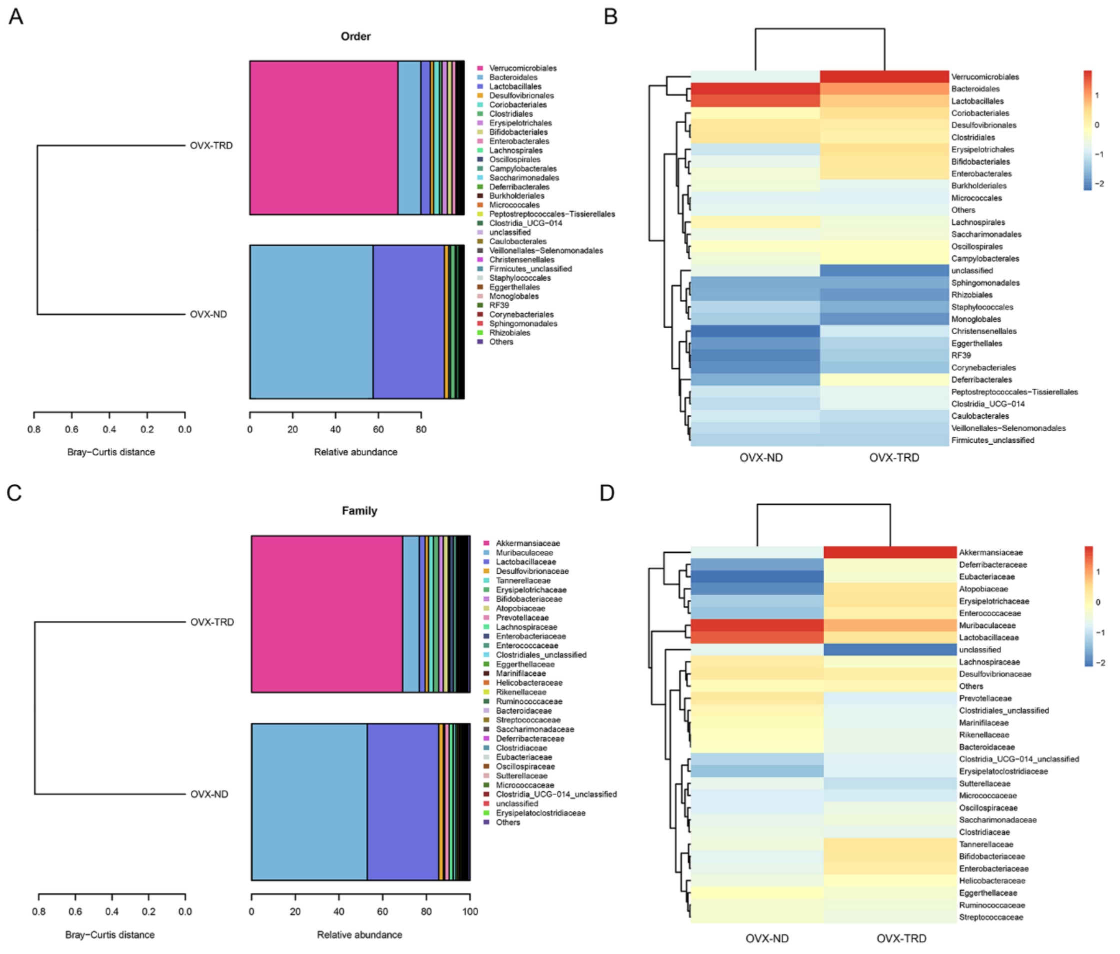

Figure S4: Intermittent tryptophan restriction shifted gut microbiota composition

at the order and family taxonomic level. (A) Bar graph of the bacteria at the order taxonomic level. **(B)** Heatmap depicting different gut microbiota at the order taxonomic level. **(C)** Bar graph of the bacteria at the family taxonomic level. **(D)** Heatmap depicting different gut microbiota at the family taxonomic level.

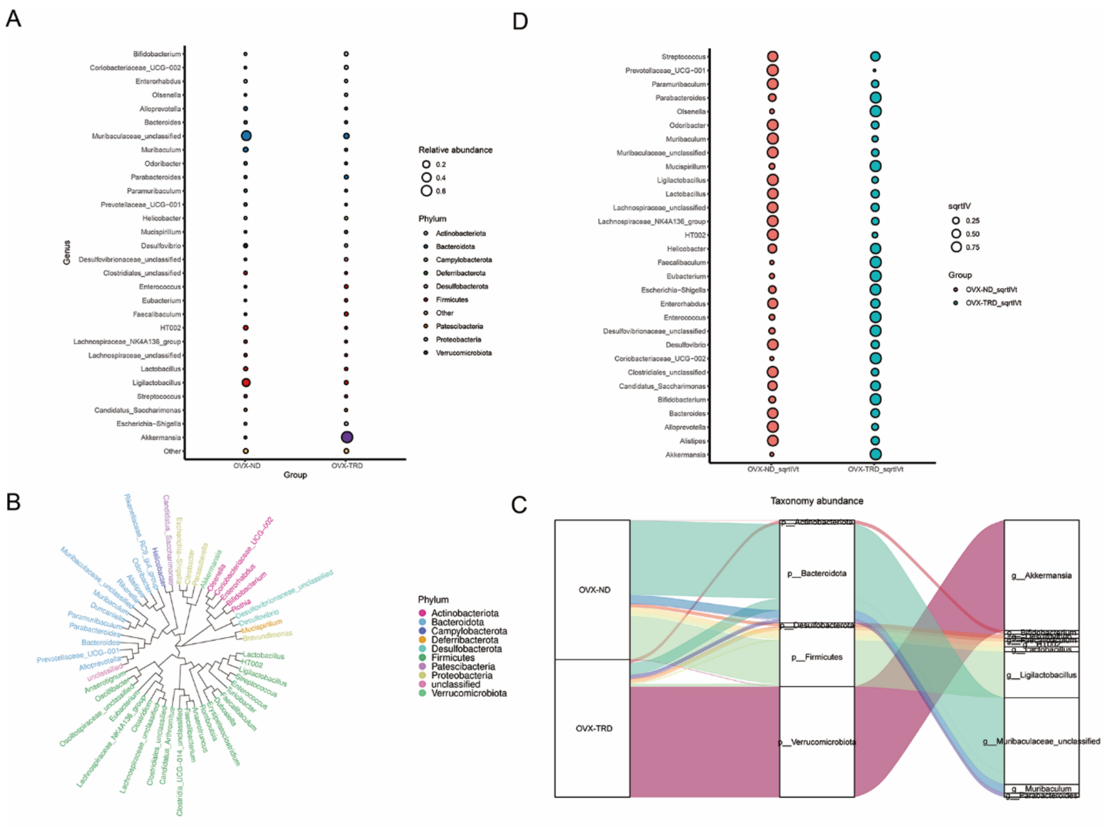

Figure S5: Intermittent tryptophan restriction shifted gut microbiota composition at the genus taxonomic level. (A) Bubble plot of the bacteria at the genus taxonomic level. **(B)** The evolutionary branching tree of the gut microbiota. **(C)** Sankey diagram of genus-level and phylum-level taxonomic shifts between the OVX-ND group and OVX-TRD group. **(D)** Bubble plot highlighting microbiome biomarkers with significant indicator values at the genus taxonomic level.

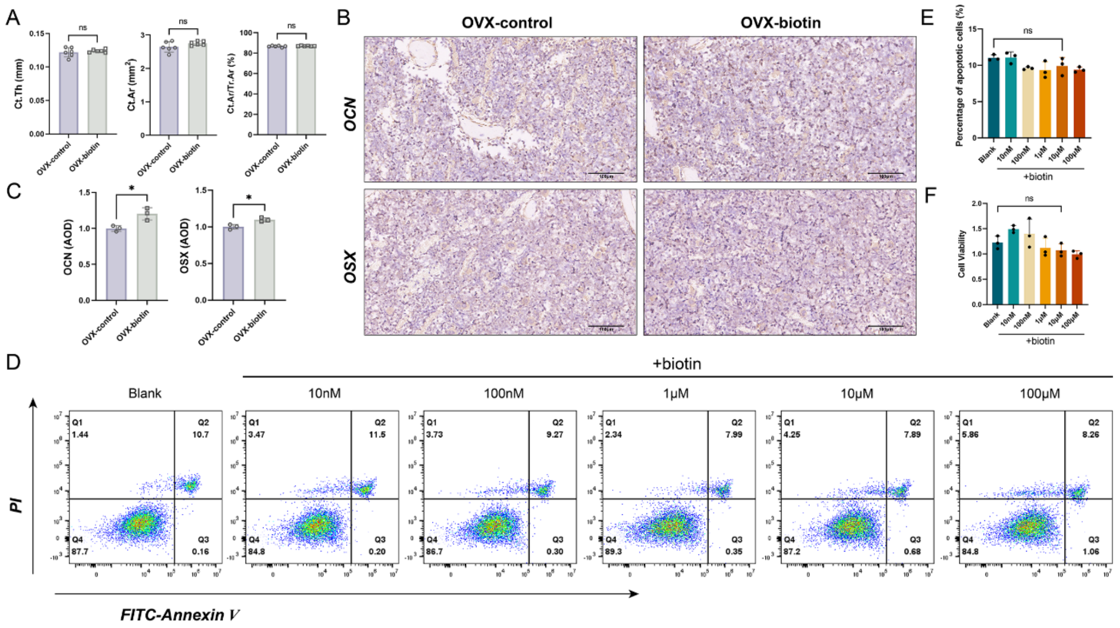

Figure S6: Biotin supplementation promoted osteogenic marker expression. (A) Quantitative analyses of cortical bone histomorphometry indices, including Ct.Th, Ct.Ar, and Ct.Ar/Tr.Ar in the OVX-control group and OVX-biotin group. **(B)** Immunohistochemistry staining of femur sections for OSX and OCN. **(C)** AOD quantification of OSX and OCN after normalization. **(D)** BMDMs were treated with biotin at 10 nM, 100 nM, 1 μM, 10 μM or 100 μM for 24 h, and apoptosis was quantified by Annexin V-FITC/PI flow cytometry. **(E)** Flow-cytometric quantification revealed that 10 μM biotin did not increase the apoptosis of BMDMs (p>0.05, ns). **(F)** CCK-8 assay confirmed that 10μM biotin exerted no cytotoxic effect on BMDMs (p>0.05, ns).

Table S1: Primer sequence for qPCR.

	Forward Primer（5’ → 3’）	Reverse Primer（5’ → 3’）
CD86	TCCTCAAACGTATTGGAAGGAGA	GCTAGGCTGATTCGGCTTCT
TNF-α	ATGAGAAGTTCCCAAATGGC	CTCCACTTGGTGGTTTGCTA
IL-1β	CACAGCAGCACATCAACAAG	GTGCTCATGTCCTCATCCTG
Gapdh	GGTTGTCTCCTGCGACTTCA	TGGTCCAGGGTTTCTTACTCC
